# Text Mining the Literature to Inform Experiments and Rationalize Impurity Phase Formation for BiFeO_3_

**DOI:** 10.1021/acs.chemmater.3c02203

**Published:** 2023-12-29

**Authors:** Kevin Cruse, Viktoriia Baibakova, Maged Abdelsamie, Kootak Hong, Christopher J. Bartel, Amalie Trewartha, Anubhav Jain, Carolin M. Sutter-Fella, Gerbrand Ceder

**Affiliations:** † Department of Materials Science & Engineering, University of California, Berkeley, California 94720, United States; ‡ Materials Sciences Division, Lawrence Berkeley National Laboratory, Berkeley, California 94720, United States; § Energy Technologies Area, Lawrence Berkeley National Laboratory, Berkeley, California 94720, United States; ∥ Material Science and Engineering Department, King Fahd University of Petroleum and Minerals (KFUPM), Dhahran 31261, Saudi Arabia; ⊥ Interdisciplinary Research Center for Intelligent Manufacturing and Robotics, KFUPM, Dhahran 31261, Saudi Arabia; # Chemical Sciences Division, Lawrence Berkeley National Laboratory, Berkeley, California 94720, United States; ¶ Department of Materials Science and Engineering, Chonnam National University, Gwangju 61186, Republic of Korea; ∇ Department of Chemical Engineering and Materials Science, University of Minnesota, Minneapolis, Minnesota 55455, United States; ○ Energy and Materials, Toyota Research Institute, Los Altos, California 94022, United States; ⧫ Molecular Foundry Division, Lawrence Berkeley National Laboratory, Berkeley, California 94720, United States

## Abstract

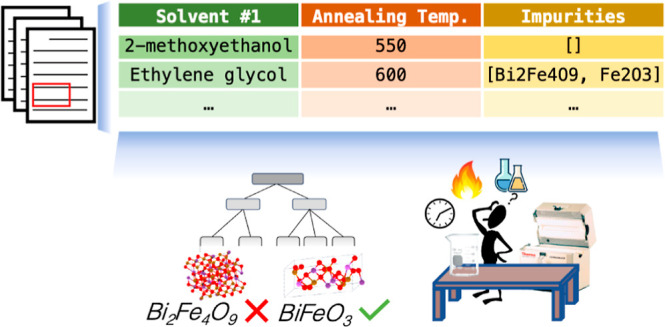

We used data-driven methods to understand the formation of impurity phases in BiFeO_3_ thin-film synthesis through the sol–gel technique. Using a high-quality dataset of 331 synthesis procedures and outcomes extracted manually from 177 scientific articles, we trained decision tree models that reinforce important experimental heuristics for the avoidance of phase impurities but ultimately show limited predictive capability. We find that several important synthesis features, identified by our model, are often not reported in the literature. To test our ability to correctly impute missing synthesis parameters, we attempted to reproduce nine syntheses from the literature with varying degrees of “missingness”. We demonstrate how a text-mined dataset can be made useful by informing new controlled experiments and forming a better understanding for impurity phase formation in this complex oxide system.

## 1 Background and Introduction

At its core, the design process for synthesizing inorganic materials, like many design processes, encompasses iterations of experiment planning, execution, and characterization of the outcome.^1,2^ The choice of synthesis conditions is critical for realizing new materials but is often made by analogy to similar materials and with limited quantitative motivation. This choice is further complicated by the expansiveness of the condition space, especially when prefiring steps (e.g., mixing, stirring, and chelation) are considered. A thorough understanding of how the choice of conditions may influence a synthesis would allow one to realize which precursors and reagents could be used at which conditions in order to achieve the appropriate evolution of phases toward a desired target.^3^ Recent studies have shown how computed thermochemical reaction energies can be used to understand synthesis pathways;^4−7^ however, these studies focus primarily on precursor choice and temperature as the fundamental conditions of interest, whereas many additional conditions are known to be relevant to inorganic synthesis.^8,9^ As the number of these conditions grows, the dimensionality of this problem increases, and it becomes harder to model the effects of these conditions on synthesis pathway determination.

Data-driven methods in synthesis prediction have the advantage of capturing the effects of features in very high dimensional spaces, a task that is difficult for humans. A significant bottleneck in this effort is the acquisition of sufficient data. The rapid gathering of relevant synthesis data can be accomplished directly through autonomous, high-throughput synthesis,^10−13^ where a synthesis machine learns optimal synthesis conditions for a specific target material or property by taking patterns from historical syntheses and their results into account. Existing studies show the promise of autonomous synthesis in accelerating the drive toward efficient materials discovery, though there are still pitfalls such as a need for condition initialization and informing experiments based on historical data.^14^ These autonomous setups can be directed by historical datasets of existing syntheses, such as through a review of reported syntheses in the scientific literature, where a wealth of historical syntheses and their detailed conditions have already been reported.

State-of-the-art natural language processing (NLP) tools make the process of text mining from the literature achievable on a large scale, without the need for a coalition of human annotators, and such methods have recently been applied to the materials science literature.^15−22,22−33^ Most of the efforts in this subfield have been in the extraction of relevant material entities, such as chemical formulas, material properties, and processing conditions; meanwhile, efforts on large-scale text mining of synthesis pathways remain limited, largely since most studies in text mining synthesis tend to assume pure target formation, without the consideration of incomplete reactions or reactions that form persistent impurity phases. Exploratory synthesis, on the other hand, rarely yields phase-pure targets. Instead, precursors or intermediate phases may persist to the end of the reaction, or the target may form and then partially decompose, leaving behind impurity phases. These impurities often have deleterious effects on the material performance but can also give insights into underlying reaction mechanisms. Unfortunately, descriptions of “failed” experiments (e.g., those that do not achieve a pure target) are rare in the published literature. There is also the problem of incomplete descriptions of procedures, which hinder meaningful modeling of the effects of the synthesis conditions and make faithful reproducibility studies difficult. Nonetheless, experimental articles mentioning impurity phase formation do exist, and with enough collected, one may be able to impute such missing parameters and, ultimately, construct a meaningful model of impurity phase formation as a function of relevant synthesis conditions. We approach such a task in this work using BiFeO_3_ (BFO) as a case study.

BFO is a promising multiferroic material with applications in spintronics as well as photovoltaic and memory devices.^34−36^ BFO in bulk has been synthesized as early as the 1960s.^37,38^ It is commonly synthesized in the nanoparticle form via either solid state^39^ or the sol–gel technique,^40^ or in thin-film form via sol–gel^41^ or physical vapor deposition.^42^ Sol–gel is a low-cost and scalable approach to synthesize thin films that are important in device industries, making it attractive for commercialization. Reports of the synthesis of BiFeO_3_ thin films emerged in earnest between 2003 and 2006.^43−45^ As in other synthesis methods, impurity phases are common in sol–gel-derived BiFeO_3_ thin-film synthesis, including iron-rich Bi_2_Fe_4_O_9_^46^ as well as bismuth-rich Bi_25_FeO_39_^47^ and Bi_25_FeO_40_.^48^ Synthesis choices that avoid the formation of these phases largely rely on heuristics. For instance, a handful of studies highlight the effect of annealing temperature on the final phase composition in this sol–gel setting,^47,49,50^ generally indicating that BiFeO_3_ has a rather narrow stability window that avoids impurity phase formation between 500 and 650 °C; this narrow stability is consistent with the results from computational work.^51,52^ Additionally, it has been shown that a Bi/Fe > 1 ratio is helpful to avoid bismuth loss, but bismuth in excess higher than 10% may lead more frequently to Bi-rich secondary phases.^53^ Although methods for synthesizing phase-pure BiFeO_3_ are known,^54,55^ understanding of the fundamental mechanisms governing the interplay between synthesis conditions and impurity formation remains limited.

**Figure 1:**
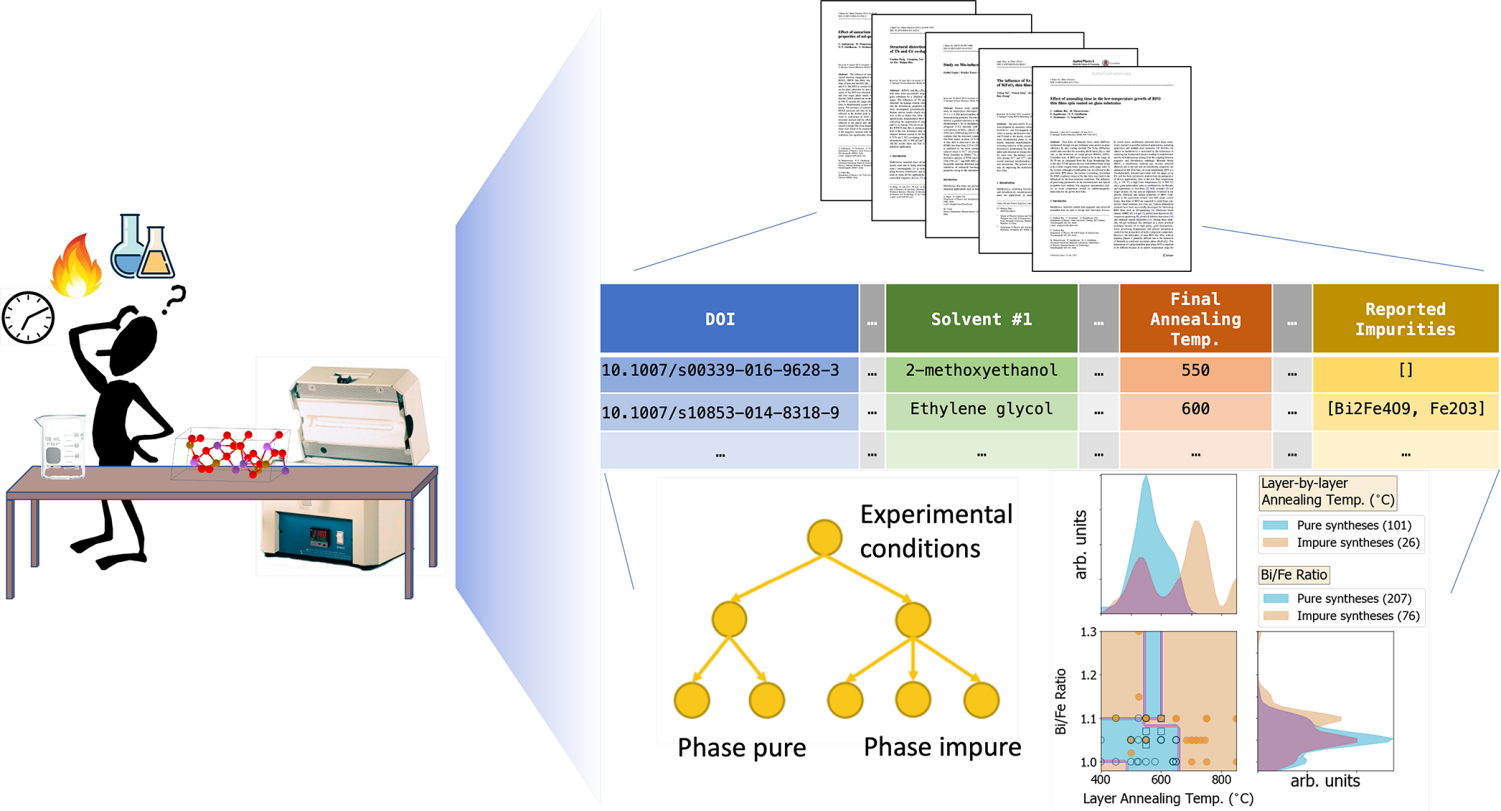
Visual summary of text mining to aide material synthesis. Left: schematic depiction of design choices for sol–gel-derived BiFeO_3_ thin films, including choices of solution precursors and reagents as well as heating and timing conditions; the final result is desired to be phase-pure, as would be indicated by phase identification in X-ray diffraction. Right: opportunities presented by text mining such synthesis procedures, including the development of predictive models (decision tree at the bottom left) or convenient visualizations of reported synthesis conditions (pairwise distribution visualization at the bottom right).

With the goal of machine learning the effects of synthesis conditions on the formation of competing impurity phases, we manually compiled a dataset of 331 synthesis procedures and outcomes from 177 articles describing the sol–gel synthesis and resulting phase content of BiFeO_3_ thin films. This aim is illustrated in Figure 1. Using these data, we trained decision tree classifiers and find that these confirm known heuristics for impurity phase formation. The models indicate that two of the most important determinants for phase impurity formation are annealing temperatures outside the window of around 500 and 650 °C and Bi/Fe metal ratios greater than 1.1 or less than 1.0, which is in line with known heuristics in the field. Feature importance analysis shows that several features related to the precursor solution preparation, such as the Bi/Fe ratio and mixing conditions, are strong predictors of phase purity. However, statistical analysis of the dataset shows that several of these features are often missing from publications, between 21 and 47% of the time depending on the condition. We conducted a set of nine experimental syntheses aimed at replicating procedures from the published literature with varying degrees of “missingness” of synthesis conditions and show to what extent missing synthesis values can be hypothesized from the body of literature. Additionally, we discovered noticeable gaps in the synthesis condition space covered by the dataset, which led us to conduct 12 new syntheses that navigate previously unexplored regions of the synthesis condition space. This modeling-experiment interaction represents a single generation in a potential active learning cycle for synthesis prediction between modeling from text mining and real experiments.

## 2 Methods

### 2.1 Text Mining and Modeling

#### 2.1.1 Compiling BiFeO_3_ Synthesis Corpus

To supply sufficient data for this text mining study, we first performed a keyword search over a database of full-text materials science articles to identify syntheses with frequent discussion of impurity phase formation. This search was performed over a body of nearly 5 million materials science publications that were scraped and parsed from online publishers, including Elsevier, Wiley, the Royal Society of Chemistry, Nature Publishing Group, the American Institute of Physics, Springer, the American Chemical Society, the American Physical Society, and the Electrochemical Society, with journals specific to materials science identified manually. Details on this process are described in a study by Kononova et al.^16^ Only articles published after the year 2000 and that are in the HTML/XML format were scraped and parsed because PDFs (comprising the majority of article formats prior to 2000) are difficult to accurately parse for scientific writing (e.g., complex chemical formulas), even through state-of-the-art optical character recognition methods.^56^ We performed a regular expression^57^ search over the full text of every paper for phrases and vocabulary related to phase purity (e.g., “impurity phase”, “secondary phase”, “phase-pure”, etc.) via an Apache Solr-based full-text search tool developed in-house (described in a study by Cruse et al.^18^). This search yielded 82,196 articles. Of these 82,196 articles, Chemical Named Entity Recognition^15^ was applied to the abstracts to extract any chemical names or formulas, under the assumption that a synthesized material of interest would be mentioned in the abstract. After normalizing the extracted names (e.g., through name-to-formula mapping, correcting for various spellings, etc.), we determined BiFeO_3_, SrTiO_3_, and LiFePO_4_, to be the most frequently discussed, with 966, 680, and 659 articles, respectively, and selected impurity phase formation for BiFeO_3_ as our focus area. Since the mechanisms for impurity phase formation vary across synthesis method and desired morphology, we narrowed our study to BiFeO_3_ thin films synthesized through the sol–gel method. Of the 966 BiFeO_3_ articles extracted above, 328 were determined to be related to sol–gel synthesis based on a previously developed synthesis paragraph classifier.^58^ Of those 328, 121 were manually determined to be related to sol–gel synthesis of BiFeO_3_ thin films and contain enough synthesis information to be suitable for the dataset (the remaining articles were related to the sol–gel synthesis of nanoparticles, synthesis of doped BiFeO_3_ thin films, or contained relevant synthesis protocols but contained no phase characterization in the text). To supplement this set, we performed a search over Clarivate Analytics’ Web of Science, specifically to supply more data for articles published after 2020 (the most recent large-scale scrape for our database). This supplementary search yielded an additional 57 relevant articles, totaling 178 articles for sol–gel-derived BiFeO_3_ thin-film synthesis.

#### 2.1.2 Extraction of Published Sol–Gel Route Thin-Film Syntheses

We manually extracted 340 sol–gel synthesis procedures from 178 papers in our corpus. We removed nine procedures that lead to an amorphous product, leading to a final dataset of 331 procedures from 177 papers. The sol–gel synthesis protocols described in the text are often very complex, spanning many values for a given condition (e.g., if the authors are studying the effects of various annealing temperatures). Additionally, the phase purity characterization is most frequently reported in a separate paragraph from the synthesis description, which makes the automatic connection of the codified recipe to the appropriate phase purity outcome difficult. Because of these challenges and the relatively small number of collected synthesis articles, the dataset of synthesis conditions and phase purity results constructed for this work was developed manually. This was accomplished by two human experts in text and data mining, machine learning, and materials science who read each paper (and supporting information, if necessary) individually for the relevant synthesis conditions and outcomes. The conditions extracted include the choice of precursor, names of solvents, chelating agents, and other reagents, spin-coating speeds and times, and the combination of temperatures and times for various heating steps (discussed in more detail in Section 3). These consisted of a total of 50 synthesis features. The outcome for each experiment was represented as a list of any specific impurity phases that formed. The full schema for the extracted dataset is given in the Supporting Information (Table S1).

#### 2.1.3 Data Processing

For data visualization and modeling, we assigned numerical values to all the features in the BiFeO_3_ sol–gel synthesis recipes dataset. After all processing, the dataset consists of 47 unique features. These processing steps are summarized below:

##### 2.1.3.1 Reported Impurity Phases

For our various modeling frameworks, we implemented both binary (0 ⇒ “phase-pure” vs 1 ⇒ “phase-impure”) and multilabel (0 ⇒ “phase-pure” vs 1 ⇒ “Fe-rich impurity” vs 2 ⇒ “Bi-rich impurity” vs 3 ⇒ “both kinds of impurity”) encodings of the reported impurity phases.

##### 2.1.3.2 Bi and Fe Precursors

The majority of published syntheses of BiFeO_3_ thin films through the sol–gel route use nitrate precursors as the Bi and Fe sources. We used label encoding where 1 indicates that nitrate precursors are used for both Bi and Fe sources, 0 indicates that either the Bi or Fe source is not from a nitrate, and −1 indicates that both Bi and Fe source are not nitrate-based.

##### 2.1.3.3 Chemical Embeddings

Because of the complex nature of sol–gel synthesis, it is important to retain as much information about the chemicals involved as possible. One-hot encoding of these components to the synthesis is not satisfactory for dimensionality reduction or modeling purposes because two chemicals would be treated as orthogonal entities, even though their function in the synthesis may be more or less similar to one another. An NLP-inspired method for capturing this similarity or dissimilarity in chemicals is mol2vec.^59^ We implemented the trained and published embedding model provided by Jaeger et al.^59^ for our purpose, which was trained over a corpus of amino acids and organic molecules. The trained embeddings contain 300 dimensions; therefore, to reduce this dimensionality, we performed principal component analysis (PCA) over the embeddings of the set of possible chemicals in this material space, leading to 61 principle components. To determine the appropriate number of PCA components that does not lead to redundant representations but compresses the data as much as possible, we investigated the convergence of pairwise cosine similarity between every chemical from 0 to 61 principal components. Based on this convergence, a reasonable number of principal components was determined to be 15. Although this is still a high number of features for one synthesis component, we find it suitable for compressing the larger data representation while maintaining sufficient fidelity. Convergence details and a table of related cosine similarities for these chemical representations are provided in the Supporting Information (Section S2).

##### 2.1.3.4 Substrate Choice

The choice of the substrate, which is largely driven by the device application, has an effect on the nucleation site preference and thus final phase homogeneity and purity. The majority of substrates in our dataset consist of Pt/Ti/SiO_2_/Si, tin-based, or glass substrates. We represent substrate choice using one-hot encoding with a separate column representing each of the aforementioned types, one column for choices other than these three, and one column for a missing substrate description. A plot depicting the most common substrates used in the dataset is provided in our Supporting Information, Figure S4.

##### 2.1.3.5 Separate Hydrolysis

Some reports^60,61^ specify the importance of mixing the bismuth and iron nitrate precursors separately in the solvent due to their different hydrolysis rates. For this, we used binary encoding to indicate whether the bismuth and iron precursors underwent separate dissolution.

##### 2.1.3.6 Annealing Atmosphere

According to the extracted data, the most typical annealing atmospheres for BiFeO_3_ thin films via sol–gel are air, oxygen, and nitrogen, with one study using argon. One-hot encoding was used to represent the annealing atmosphere.

##### 2.1.3.7 Filling in Missing Data for Exploratory Data Analysis

To deal with missing values in our dataset prior to exploratory data analysis, we set all remaining quantitative values to 0 if they were not provided.

##### 2.1.3.8 Filling in Missing Data for Modeling

For modeling purposes, it is necessary to impute missing data that would be necessary to replicate the synthesis (e.g., precursor concentration, Bi/Fe ratio) or implied to exist but were simply not provided (e.g., a prebake step was used and the temperature was given, but the time was not). More details on the frequency of such missing information are provided in Section 4.1 and the Supporting Information (Figure S5). There are many techniques available to impute such data, known as missing value imputation (MVI) methods. In our study, we implement the most popular statistical (substituting median values) and machine-learned (*k*-nearest neighbors) imputation methods according to reviews of MVI methods.^62,63^ For *k*-nearest neighbors imputation, we found *k* = 5 to be an adequate number of neighbors to consider when imputing missing values, based on an analysis of imputing randomly masked values in our dataset. Details of this analysis are given in Section S4.2 in the Supporting Information.

#### 2.1.4 Decision Tree Modeling

We have sought to train an interpretable machine learning model that provides insights into the effects of synthesis conditions on the formation of impurity phases. While there are many classifier algorithms available with robust predictive power, we found that more advanced models perform similarly to the decision tree on our text-mined dataset. Because of this performance similarity and our prioritization of easily interpretable predictions, we decided to move forward with the decision tree classifier for this task, using the sci-kit learn’s Decision Tree Classifier module (https://scikit-learn.org/stable/modules/tree.html). Details of our comparison of different classifiers are provided in our Supporting Information Section S5.

In our comparison, we considered four model frameworks using a different combination of MVI (between median value and *k*-nearest neighbors imputation) and prediction scheme (between binary and multilabel). For evaluation, we only considered the binary prediction task: phase-pure vs phase-impure. Within each of these frameworks, we considered 10 different randomized splits for training and testing data, with 20% of the data held out for testing in each, resulting in 40 possible models. In each of these splits, the appropriate hyperparameters were determined through cross-validation. The best estimator from each split was then applied to the held-out test data to obtain the evaluation metrics. Details on the hyperparameters of interest and the various evaluation metric values are given in Section S5.1. Model comparison showed comparable performance between all models, as shown in Section S5.2. Because we prioritize easy interpretability, we decided to move forward with a simple decision tree model. With our best-performing decision tree models, we constructed learning curves from each of these 40 estimators by fitting the models to an increasing number of training samples (from 10 to 80% of the total dataset).

### 2.2 BiFeO_3_ Synthesis Experiments

#### 2.2.1 Film Fabrication

Experimental syntheses were performed either for exploratory purposes or as an attempt to replicate one of the four published procedures.^46,64−66^ Precursor solutions were prepared by dissolving Bi(NO_3_)_3_·5H_2_O(≥99%, Sigma-Aldrich) and Fe(NO_3_)_3_·9H_2_O (≥99%, Sigma-Aldrich) in 2-methoxyethanol [2-ME] (anhydrous, 99.9%, Sigma-Aldrich). The stoichiometry of Bi/Fe was varied between 0.9 and 1.05 depending on the experiment. For films prepared with a chelating agent, citric acid (99.9%, Sigma-Aldrich) was added to the precursor solution with a molar ratio of citric acid/metal salt (4:1). Stirring temperatures and times were between 25–90 °C and 2–24 h, respectively, depending on the experiment. Each solution had a concentration of either 0.25 or 0.4 M depending on the experiment. After complete dissolution of the precursors, the solution was spin-coated on either glass substrates, or the relevant substrate if the experiment is an attempt to replicate a published synthesis (see Section 4.3), at 3000 rpm for 30 s. Then, the sample was dried on a hot plate at 80 or 200 °C for 2 or 10 min and baked on a hot plate at 350 or 400 °C for 5 min according to the experiment. All drying and baking was performed in air. The spin coating/baking procedure was repeated five times to obtain thick films. The as-cast baked films were annealed in a tube furnace at 550 or 640 °C with various annealing times for exploratory experiments or at the reported temperature for replication experiments. Film preparation was performed in air or O_2_ atmosphere depending on the experiment.

##### 2.2.2 X-ray Diffraction

XRD measurements were performed at room temperature in the 2θ range of 10–60° with a step size of 0.01° and a scan speed of 4° min^–1^, using an X-ray diffractometer (Rigaku, SmartLab) with Cu Kα radiation (1.5406 Å) and a HyPix-3000 high-energy-resolution multidimensional semiconductor detector.

## 3 Typical Sol–Gel-Derived BiFeO_3_ Thin-Film Synthesis

**Figure 2:**
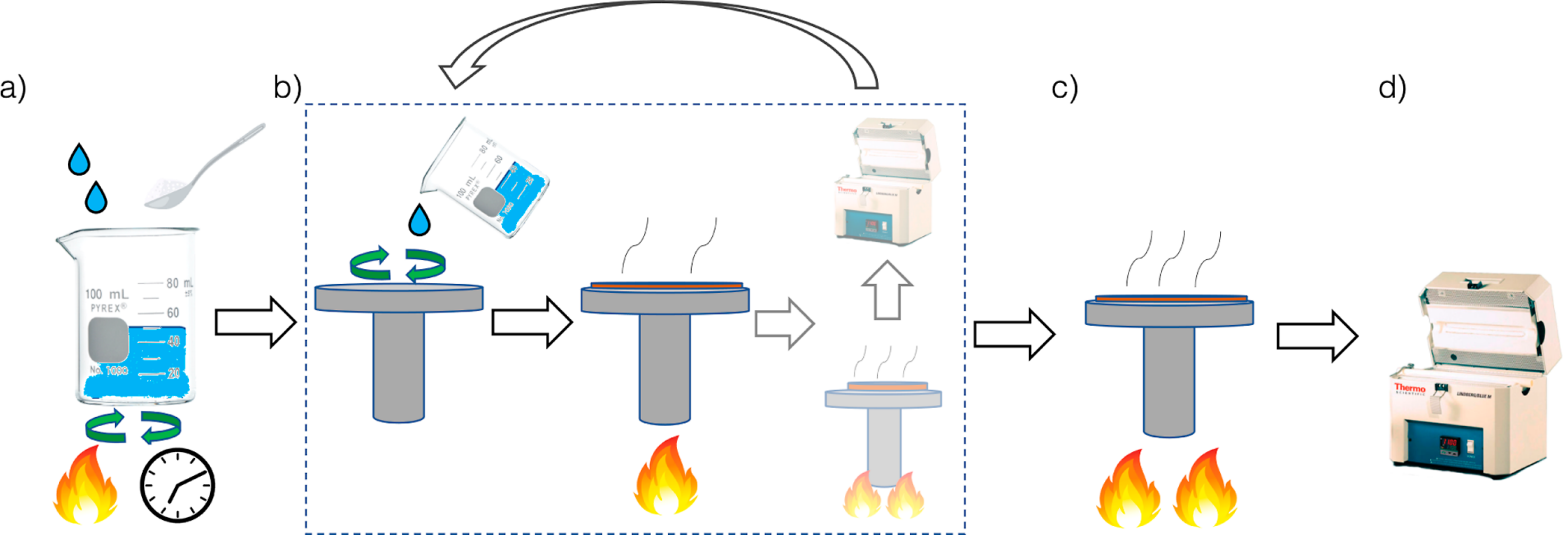
Schematic of typical sol–gel BiFeO_3_ thin-film synthesis. (a) Metal precursor salts are mixed with solvents and other reagents under choice of heating and stirring time. (b) Mixed solution is deposited onto a spinning substrate, usually in multiple layer-by-layer steps to reach the desired film thickness; the repetitive layer-by-layer coating procedures can be applied either after drying, prebaking, or annealing steps. (c) Optional pyrolysis (or prebake) step to remove any remnant organic species. (d) Annealing to crystallize the final phase.

To provide context for this synthesis space and its existing heuristics, we provide a walkthrough of a typical sol–gel BiFeO_3_ synthesis. As discerned from the dataset constructed for this work and a review of sol–gel BFO thin-film synthesis by Zhang et al.,^41^ the primary steps of such syntheses include: (a) solution (“sol”) preparation and gelation (“gel”), (b) deposition and spin-coating along with possible drying and pyrolysis steps, (c) postdeposition pyrolysis, and (d) the final crystallization (see Figure 2). Details of each step depicted in Figure 2 are described below.

(a) The preparation of the solution involves mixing metal salt precursors with a solvent and possible chelating agent. Bi and Fe precursors are most typically nitrate-based, though several studies (particularly early sol–gel thin-film syntheses) use acetate-based precursors. The Bi precursor is often added in excess of the Fe precursor due to the volatility of bismuth metal during annealing.^67^ Typical solvents include 2-methoxyethanol and ethylene glycol or a combination of the two. Chelating agents such as citric acid, acetic acid, and acetic anhydride are frequently used in solution preparation since they balance the rates of hydrolysis and condensation of metal–organic complexes, which aides in the formation of the ultimate “gel” without unwanted precipitation.^41^ During mixing of the solution components, the mixture may be heated beyond room temperature to improve homogeneity, particularly if solid citric acid is used as a chelating agent. The solution may be aged on the order of days prior to deposition.
(b) The prepared solution is then deposited onto a substrate, which is spun to create an evenly coated layer. The spinning step may include only one step or two (the second step having a higher spinning rate than the first). The process should be repeated several times in order to reach the desired thickness. The thin film is then often dried, which takes place at temperatures of ∼100 °C.
(c) Pyrolysis (prebaking) may be included to remove extraneous solvent and organic material. This occurs either after all layers are spun onto the substrate or between every layering step (“layer-by-layer pyrolysis”, depicted by the greyed out portion in Figure 2b). This typically takes place at slightly higher temperatures (∼300 °C).
(d) Annealing for crystallization is the highest temperature step (generally between 500 and 600 °C). This may be executed once after all layers are deposited or between every layering step (“layer-by-layer annealing”, depicted by the grayed out portion in Figure 2b). Sometimes, experimentalists use a different temperature during the final annealing compared to the layer-by-layer steps.

The phase composition of the final sample is then determined using XRD. Impurity phases are often detected in the final film, including the binary oxides Bi_2_O_3_ and Fe_2_O_3_, Fe-rich Bi_2_Fe_4_O_9_, and several Bi-rich phases such as Bi_25_FeO_30_, Bi_25_FeO_40_, and Bi_36_Fe_24_O_57_. The mullite Bi_2_Fe_4_O_9_ and sillenite Bi_25_FeO_39_ (and other related) phases are known to be thermodynamically competitive with the target BiFeO_3_ phase.^68^ Hypotheses for the mechanisms leading to the formation and frequent persistence of these two phases have been investigated previously for solid-state settings, including the possibility of competing diffusional processes^39^ and pseudo-ternary phase competition between the starting bismuth and iron precursors and metal oxide impurities present in those precursors.^69^ For sol–gel synthesis, in-depth studies on the chemical processes encountered in the precursor solution have been conducted;^70^ however, mechanistic understanding for the formation of these impurity phases in wet chemical environments (such as in sol–gel synthesis which is the focus of this study) is largely driven by analogy or extrapolation from these solid-state studies.

## 4 Results

We divide the results of our study into four sections: (1) a summary of the conditions extracted in our text-mined synthesis dataset, (2) results from predictive modeling of impurity phase formation using decision trees, and results from informed experiments focused on (3) reproducing existing results, and (4) exploring underexamined synthesis condition spaces.

### 4.1 Text-Mined Dataset

**Figure 3:**
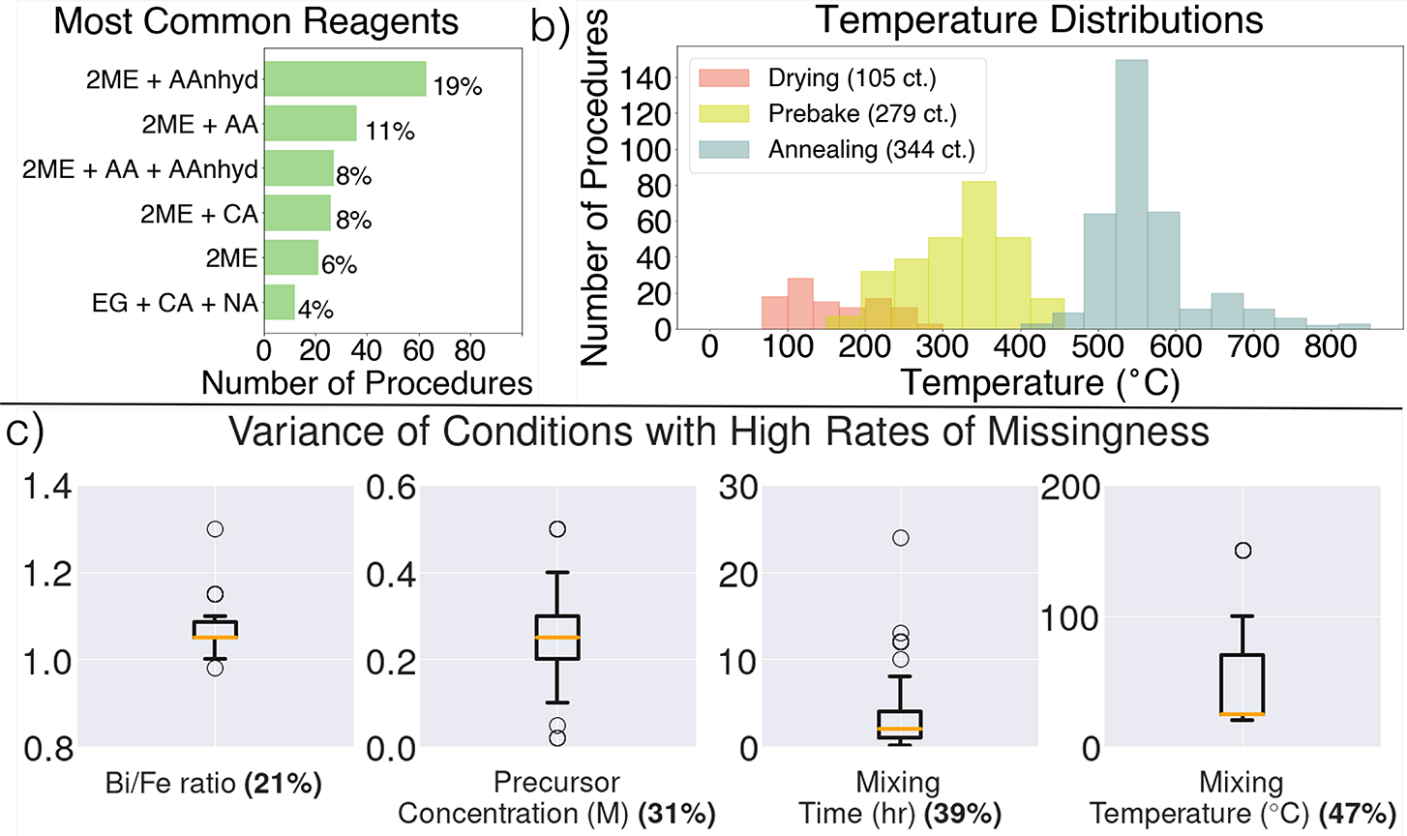
Summary of reported synthesis conditions. (a) Most frequently used combinations of solvents and chelating agents (out of 331 synthesis procedures); 2ME = 2-methoxyethanol, AAnhyd = acetic anhydride, AA = acetic acid, CA = citric acid, EG = ethylene glycol, and NA = nitric acid. (b) Histograms for drying, prebake, and annealing temperatures. (c) Box and whisker plots for the values of the missing synthesis conditions that are considered necessary for proper replication of a procedure and which are most frequently missing; orange lines indicate median value, bottom and top box boundaries are first and third quartiles, respectively; whiskers represent 1.5× extension from quartile bounds; individual points represent outliers; and bold percentages in *x*-axis indicate the percentage of articles missing that condition.

Across all 331 extracted experiments, 24.2% resulted in a sample containing one or more impurity phases; across all 177 articles, 21.4% contained at least one experiment resulting in phase impurities. The most commonly appearing impurity phase is the Fe-rich Bi_2_Fe_4_O_9_. Several Bi-rich phases, such as Bi_25_FeO_30_, make up the next most prevalent impurity phases, followed by the binary oxides Bi_2_O_3_ and Fe_2_O_3_. The overwhelming majority of syntheses (317 out of the 331 extracted syntheses) use hydrated nitrates as the metal precursors. A visual summary of common chemical reagents (solvents and chelating agents), processing temperatures, and frequently omitted information is provided in Figure 3.

The most common combinations of chemicals used to build the precursor solution are listed in Figure 3a. The most frequently used solvent is 2-methoxyethanol (“2ME”), and it is most often mixed with chelating agents such as acetic anhydride (“AAnhyd”), acetic acid (“AA”), or a combination of the two. Another common chelating agent that is sometimes mixed with 2-methoxyethanol is citric acid (“CA”). Ethylene glycol (“EG”) is used less frequently and is usually mixed with citric acid and nitric acid (“NA”).

The range of choices for temperature in the various heating steps in this synthesis process is illustrated through the histograms in Figure 3b. “Layer-by-layer” and “final” prebake and annealing steps are combined for their respective distributions, which is why the “Annealing” histogram contains more than 331 counts. Each step shows a skewed overall distribution (left-skewed for drying and annealing steps and right-skewed for prebake). Additionally, each step has a fairly substantial spread of temperatures, ranging across about 200 °C for drying steps, 300 °C for prebake steps, and over 400 °C for annealing steps. The normalized standard deviations are 0.38, 0.22, and 0.12 for the drying, prebake, and annealing steps, respectively. The extent to which samples need extensive drying and prebaking will depend on the organic reagents used and the concentration of the precursor; thus, their value will depend on those experimental choices, partly explaining why drying and prebake temperatures have larger normalized standard deviations; conversely, the annealing temperature for crystallization of the target phase is less dependent on these choices and more on an historical understanding for the typical temperatures needed to achieve a phase-pure product, in this case between 500 and 600 °C.

While the statistics for the extracted values in such synthesis choices are helpful in verifying appropriate diversity and breadth of sampling for the dataset, it is also important to understand the level of “missingness” of conditions in the procedures extracted here. In fact, many of the syntheses extracted in the dataset are missing conditions that should be considered vital to successfully reproduce the synthesis, which we highlight in Figure 3c (note that supporting information was inspected during extraction, as well). The minimum information for such reproducibility is debatable, particularly since those familiar with the field may be able to intuit certain conditions based on the total literature. For the purposes of learning synthesis directly from the literature, however, we consider the following information at a minimum to be necessary for a “complete” recipe (with any other omitted information assumed to simply not be included in the synthesis, such as aging times):

- precursors and reagents used (including metal nitrates, solvents, and chelating agents)
- precursor and reagent amounts
- Bi/Fe molar ratio
- precursor solution molar concentration
- stirring conditions (time and temperature)
- annealing conditions (time and temperature)

We acknowledge that this variable list may not be exhaustive in describing a synthesis. Of these conditions, the least frequently omitted are the metal precursor and reagent choice and the choice of annealing conditions, all of which are left out of only two articles. Figure 3c provides a statistical breakdown of the values employed for the remaining experimental conditions listed above along with their frequency of omission. The box-and-whisker plots illustrate the range of values employed for each of these conditions, along with the median value (orange line), first and third quartiles (bottom and top of boxes, respectively), 1.5× extension from the quartile bounds (whiskers), and remaining outliers (points). The bold percentages in the *x*-axis labels represent the fraction of recipes in the dataset that are missing that condition. The Bi/Fe ratio is missing from 38 articles (21%) of articles. This is an important condition to consider for these syntheses because too little bismuth may lead to bismuth loss (due to its volatility) but too much will often lead to Bi-rich impurity phases.^53^ The distribution of values for the Bi/Fe ratio is fairly concentrated around 1.05; nonetheless, studies^53,71^ have shown that even small deviations in this ratio (ΔBi/Fe ∼0.03) will affect the resulting phase composition. Precursor concentration and mixing conditions (temperature and time) all show fairly wide distributions of values, making it difficult to reliably assume the value that was used for a given synthesis if that information is omitted. The concentration of the precursor solution is missing from 54 articles (31%). This value is also important to include since the metal nitrate concentration in the solution is expected to influence the homogeneity of the coated layers, as well as the chances of unwanted precipitation during gelation.^41^ Finally, the most frequently omitted processing conditions are the time (69 articles, 39%) and temperature (83 articles, 47%) of solution stirring. These conditions are important to include since they will also determine the homogeneity of the predeposited solution, particularly when solid reagents with dissolution temperatures above room temperature, such as citric acid, are used. This overall lack of a uniform and complete synthesis procedure ontology^72^ also causes problems in modeling where missing values must either be replaced by (possibly erroneous) interpolated values or require entire data points to be removed, leading to the worse model performance. We deal with such missing values using statistical (from the median value) and machine-learning (from *k*-nearest neighbors) imputation methods in our modeling. A larger-scale visualization of the frequency of missing data is given in Figure S5.

### 4.2 Interpretable Phase Impurity Formation Modeling

To predict the formation of impurity phases based on synthesis conditions, we trained decision tree models using the text-mined dataset. Our attempts to construct an interpretable model that predicts impurity phase formation based on synthesis conditions proved to have limited performance. Nevertheless, we are still able to recover well-known heuristics for phase-pure synthesis, and we identify important determinants of impurity phase formation in the preparation of the precursor solution.

**Figure 4:**
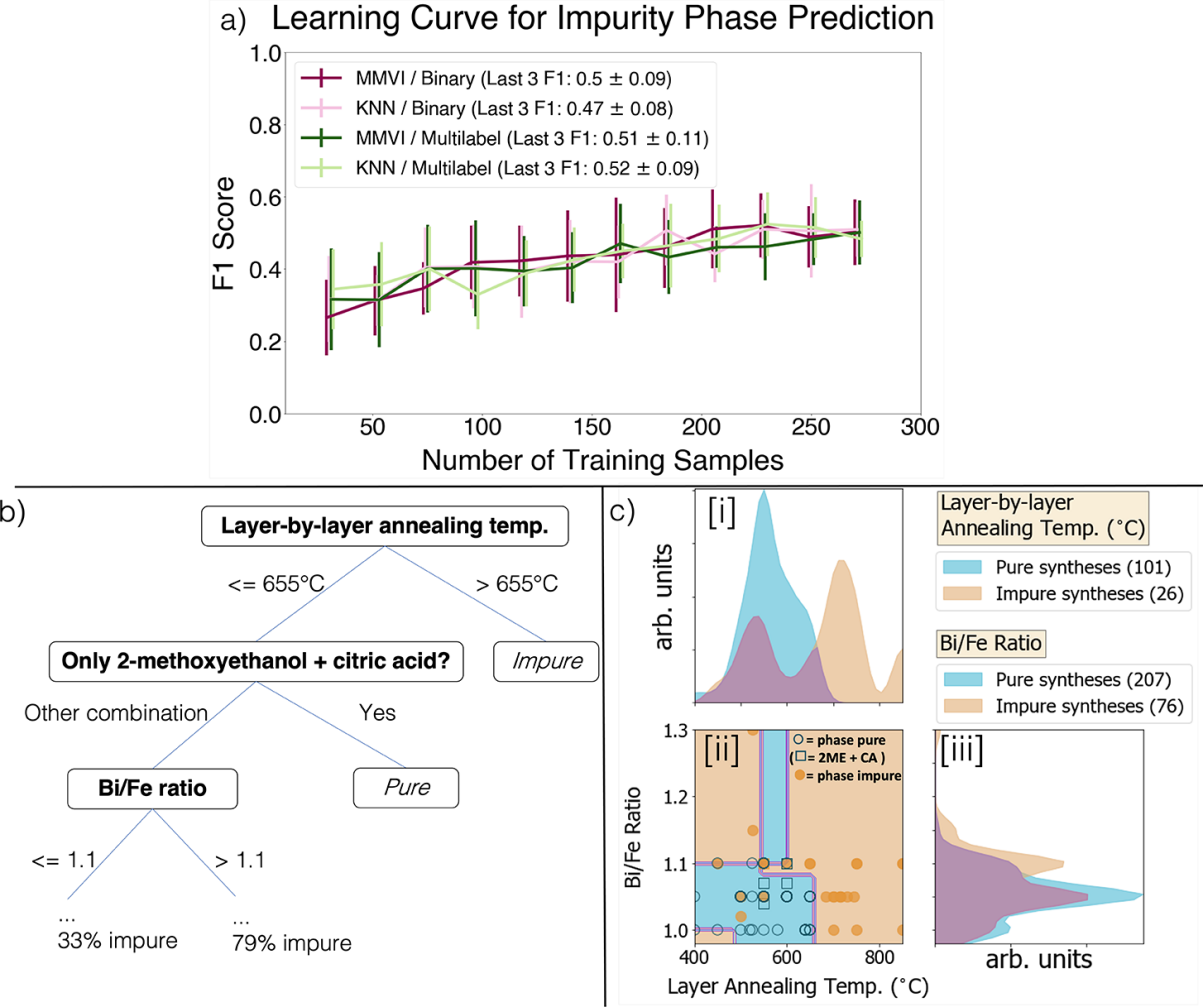
Predictive modeling of text-mined dataset. (a) F1 scores across increasing training set size (from 10 to 80% of total data, using 20% of data for test set in each evaluation). Error bars are generated through six different randomizations in train/test split for each number of training samples. Each curve represents a different combination of MVI and output labeling: “mmvi” ⇒ median missing value imputation; “*k*nn” ⇒ *k*-nearest neighbors MVI; “binary” ⇒ model predicts whether synthesis results in phase-pure synthesis or has phase impurities; “multilabel” ⇒ model predicts outcomes among “phase-pure”, “Fe-rich impurities”, “Bi-rich impurities”, and “both kinds of impurities”.(b) Root of sample decision tree trained over text-mined dataset; specific features and decisions made at each node are provided. (c) Visual representation of important numerical features used as decision boundaries in the decision tree model: layer-by-layer annealing temperature and Bi/Fe molar ratio; (c)(i) and (c)(iii) panels show the distribution of values found from the literature for each of these parameters across phase-pure (blue) and phase-impure (orange) syntheses, with crossover between these outcomes indicated in purple; smooth distributions are constructed through Gaussian kernel density estimation (KDE); and (c)(ii) panel shows decision boundaries learned by this decision tree estimator, with blue regions indicating a phase-pure sample and orange regions indicating a sample with impurity phases. A scatter plot of individual data points overlays the decision boundaries; phase-pure points are empty blue circles, phase-impure points are solid orange circles, and square points represent syntheses using 2-methoxyethanol + citric acid [corresponding to the second decision made in (b)].

We chose to model this task using a decision tree for its easy interpretability (as if training a new bench chemist how to make a set of decisions based on available resources) and ability to capture nonlinear relationships. The details for data featurization and training are described in Section 2.1.4, and the performance and inferences made by the model are summarized in Figure 4. In Figure 4a, we show the learning curves for four different model frameworks: binary classification using median imputation for missing values; binary classification using *k*-nearest neighbors imputation for missing values; multilabel classification using median imputation for missing values; and multilabel classification using *k*-nearest neighbors imputation for missing values. We did not need to consider the imputation of any categorical variables since those were frequently reported.

Because our dataset shows an imbalance between phase-pure and phase-impure syntheses (75.8%:24.2%), an appropriate evaluation metric should be chosen so as not to misrepresent the predictability of the model. Unlike traditional accuracy scores, the F1 score, which represents a harmonic mean between the precision and recall of a classifier, takes into account the false negatives and false positives (in this case, syntheses predicted to be phase-pure but actually contain phase impurities and syntheses predicted to have phase impurities but actually are phase-pure, respectively). It should be noted that the F1 score does not completely remove issues from evaluating imbalanced classification tasks, such as the fact that the class distribution during training and even testing may not persist when the model is used out-of-the-box. Nevertheless, this is a partial solution for evaluating the present task. We include evaluation on additional metrics, such as the Mathews Correlation Coefficient, in the Supporting Information.

Both multilabel models show better performance compared to their binary classifier counterparts, showing that providing more information to the model (here, what specific types of impurity phases form) helps improve the prediction ability. The variation in F1 score for all models is quite substantial but seems to improve with the addition of more data points, indicating that a larger dataset can help improve model stability further. Both MVI methods perform similarly, with *k*NN-based imputation showing slightly better variation in F1 score, highlighting a slight preference for ML-based imputation when consistency in performance is desired.

Despite the limited predictive power of these models, our best-performing models can identify decision boundaries that corroborate known experimental heuristics. Feature importance values from our modeling were tabulated and averaged across each training. The top 5 most important features were determined to be, in order, (1) the layer-by-layer annealing temperature, (2) the Bi/Fe ratio, (3) the final annealing temperature, and the (4) mixing temperature and (5) time. These rankings are in line with known heuristics in the field, as discussed in Section 2.2.1, and, incidentally, highlight the risk of not including vital synthesis details such as the Bi/Fe ratio and mixing conditions, both of which are often omitted from procedure descriptions as mentioned in Section 4.1. To assess the importance of these under-reported variables and the possible ineffectiveness of MVI, we conducted nine experiments aimed at reproducing reported syntheses with varying degrees of missingness (see Section 4.3).

We further inspect the ability of our models to distinguish important boundaries in the synthesis condition space through the top of the tree for one of these models, as shown in Figure 4b. We note that the order in which decisions appear does not necessarily reflect the order of decisions that would be made in a laboratory but rather reflect the hierarchy of importance of the features in predicting the final phase purity of the sample, as determined by the model. From the root of the tree in Figure 4b, the model first observes whether the synthesis employs layer-by-layer annealing at a temperature above (traversing to the right) or below (to the left) 655 °C. This value is not a specific value found in the dataset but rather a boundary in the 47 dimensional feature space determined by the decision tree model to most effectively discriminate between phase-pure and phase-impure syntheses. Syntheses that anneal above this temperature are decidedly impure, and those below this temperature encounter further decisions. A visualization of the values for the parameters reflected in Figure 4b along with the synthesis outcome (phase-pure or phase-impure) is shown in Figure 4c. Here, Figure 4c(i) illustrates the distribution of layer-by-layer annealing temperatures employed across the dataset, with distributions (calculated using Gaussian kernel density estimation) distinguished between phase-pure and phase-impure syntheses. The *y*-axis units are arbitrary since the distributions are scaled according to the number of samples in that subset (i.e., pure or impure). The distributions support the first decision made by the tree since we see a noticeable shift in the centers of the distributions for phase-pure syntheses and phase-impure syntheses at temperatures of 500 °C and higher. Following now the left-hand branches of Figure 4b, the next observation made by the model is whether the metal nitrates are dissolved in a mixture of 2ME and CA (traversing to the right) or some other chemical mixture (traversing to the left). The majority of syntheses in the dataset traverse the leftward path, and all of those traversing to the right result in a phase-pure sample. Upon inspection of the dataset, every procedure (27 total) that uses only 2ME and CA as its precursor solution reagents (i.e., traveling to the right from this node) results in a phase-pure sample. Samples from the text-mined dataset using this combination of chemicals are depicted as squares in Figure 4c(ii), and all of those are blue, representing phase-pure synthesis. Because this decision is made on a nondiversified subset of the dataset (i.e., every relevant data point is phase-pure), such a result presents an opportunity for hypothesis testing (e.g., “does the specific combination of 2ME + CA mitigate the formation of impurity phases more than other reagents?”) and to probe underexplored regions of the synthesis condition space. Finally, the model queries whether the Bi/Fe ratio is greater than (to the right) or less than or equal to (to the left) 1.1. Investigating the Bi/Fe molar ratio distributions for phase-pure and phase-impure syntheses in the bottom-right panel of Figure 4c(iii), we see that, indeed, a noticeable peak and tail in the distribution of phase-impure syntheses are seen at Bi/Fe ratios at and above 1.1:1.

We emphasize the overall complexity in predicting phase purity based on the provided synthesis conditions through the pairwise scatterplot between these two parameters, as shown in the bottom-left panel of Figure 4c(ii). Here, individual phase-pure (blue circles/squares) and phase-impure (orange dots) syntheses overlay the decision boundaries made by the decision tree model from Figure 4b, with orange regions representing phase-impure synthesis and blue regions being phase-pure. Compared to the single-condition distributions shown in Figure 4c(i) and Figure 4c(iii), Figure 4c(ii) makes it apparent that distinguishing regions of combinations of conditions leading with certainty to a phase-pure sample from those leading to phase impurities becomes more difficult as the number of conditions considered increases. This is particularly true for less predictive parameters, as seen in the full set of pairwise purity visualizations in Figure S6. Additionally, despite the easy interpretability of decision tree modeling, the decisions made are not always physically reasonable. For instance, the strip of blue predicting “phase-pure” syntheses above Bi/Fe ratio = 1.1 is within the typical window of phase-pure syntheses when considering annealing temperature; however, every synthesis above that Bi/Fe ratio shows phase impurities. The density of points in this region is low, so additional testing in this subspace would help improve the quality of decisions made here.

### 4.3 Reproducibility of Published Procedures

To investigate the importance of specific synthesis condition variables in reproducing published experiments, we conducted a set of nine experiments aimed at replicating the results from four separate papers. The syntheses from these papers^46,64−66^ were chosen specifically because they were missing what we believed to be vital information to the successful replication of the experiments (discussed more in Section 4.1). For these missing conditions, we substituted either median values or typical choices from the literature (particularly if a median value does not make sense for another given condition, such as stirring at room temperature while using citric acid) or we considered ranges of possible reasonable values. The conditions, results, and predictions made by our models for these are provided in Table 1.

**Table 1:**
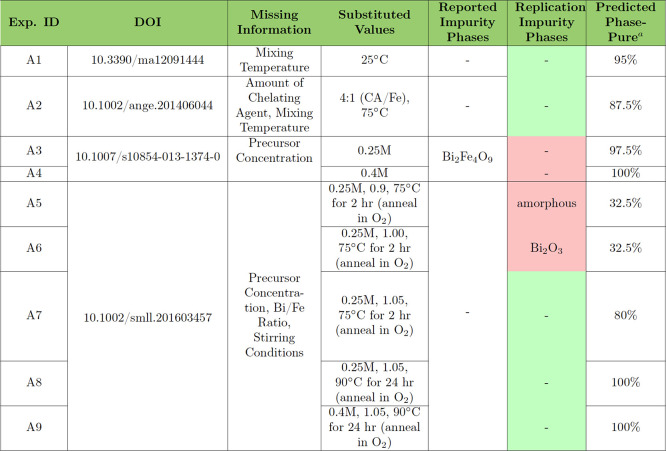
Suggested Experimental Conditions to Reproduce Experiments in the Literature with Missing Values

a Predicted outcome based on percentage of “phase-pure” predictions among the 10 best-performing models from each randomization seed and four modeling frameworks (40 predictions total).

Our attempt to reproduce the phase-pure synthesis in A1,^64^ which was missing only the mixing temperature, was successful when we substituted 25 °C, the median mixing temperature. Our best-performing models predict that this synthesis would be phase-pure 95% of the time. Inspection of the decision trees reveals that the use of 2-methoxyethanol and ethylene glycol together in the precursor solution often contributed to the 5% of phase impurity predictions. Experiment A2,^66^ which resulted in phase-pure BiFeO_3_ in the literature, was missing the amount of chelating agent (citric acid in this case) and the mixing temperature. Our replication attempt was successful when using the typical amount of citric acid chelating agent (4:1 CA/Fe) and median mixing temperature for synthesis in the literature that includes citric acid (75 °C). Our decision trees correctly predict this synthesis, leading to a phase-pure result, 87.5% of the time. Of note, this procedure combines 2-methoxyethanol and citric acid in its precursor solution, which was shown to be a useful predictor for phase purity in this dataset (see Section 4.2). A3–A4^46^ reported a Bi_2_Fe_4_O_9_ impurity phase and was missing the concentration of the precursor solution. We were unable to reproduce this result since we were only able to produce phase-pure BiFeO_3_ using either the median precursor concentration (0.25 M) or a higher-than-typical precursor concentration (0.4 M). Interestingly, our model predictions for this set returned a high percentage of phase-pure predictions, which agrees with our results but disagrees with what was originally reported. Inspection of the decision tree paths traversed for this procedure shows that the annealing temperature of 500 °C and the combination of 2-methoxyethanol with ethanolamine in the precursor solution are frequently considered as factors leading to phase-pure predictions. The high percentage of phase-pure predictions along with the lack of production of impurity phases indicates that the actual precursor concentration used may have been less than the median value contained in the text-mined dataset. Finally, A5–A9^65^ reported phase-pure BiFeO_3_ and was missing the precursor concentration, the Bi/Fe ratio, and the stirring conditions. Our first replication attempt, A5, used the median precursor concentration (0.25 M), a 0.9:1 Bi/Fe metal ratio (lower-than-typical), and the median stirring temperature and time provided in the dataset for synthesis with citric acid. This attempt resulted in an amorphous film and thus failed to reproduce the reported results. Our second attempt, A6, used the same precursor concentration and stirring conditions, but we increased the Bi/Fe ratio to 1:1. This led to a binary Bi_2_O_3_ impurity phase. We then increased the Bi/Fe metal ratio once more to the median value, 1.05:1, in A7, which successfully reproduced the reported result. We extended these trials by increasing both the stirring time and temperature (due to the limited solubility of solid citric acid, lower stirring temperatures produce an inhomogeneous precursor solution) and precursor concentration (since, according to the dataset, lower precursor concentration has a higher tendency to lead to phase impurities compared with higher precursor concentrations). Both of these attempts, A8 and A9, also produced a phase-pure target, indicating that the Bi/Fe metal ratio may be the most vital missing information in this case, which is in line with the feature importance values determined by our decision trees in Section 4.2. It should be noted that the intuition for a greater-than-one Bi/Fe ratio would require domain knowledge from a prospective experimenter attempting to replicate such a recipe; still, this points to the importance of specifying this information, particularly if the volatility of a particular precursor is not explicitly discussed. Additionally, our models predicted phase-pure syntheses much less frequently for A5 and A6 than for A7–A9, which agrees with our results where phase impurities formed only for experiments A5 and A6. The most decisive factor leading to phase-impure predictions for A5 and A6 is the lower-than-median Bi/Fe ratio. The majority of predictions made for A7–A9 were phase-pure; however, the regions of the conditions space covering these procedures are data poor, especially for A8 and A9. This local lack of diverse data inspired additional syntheses to explore these synthesis condition regions of interest, as is discussed in Section 4.4.

### 4.4 Informing New Experiments

**Figure 5:**
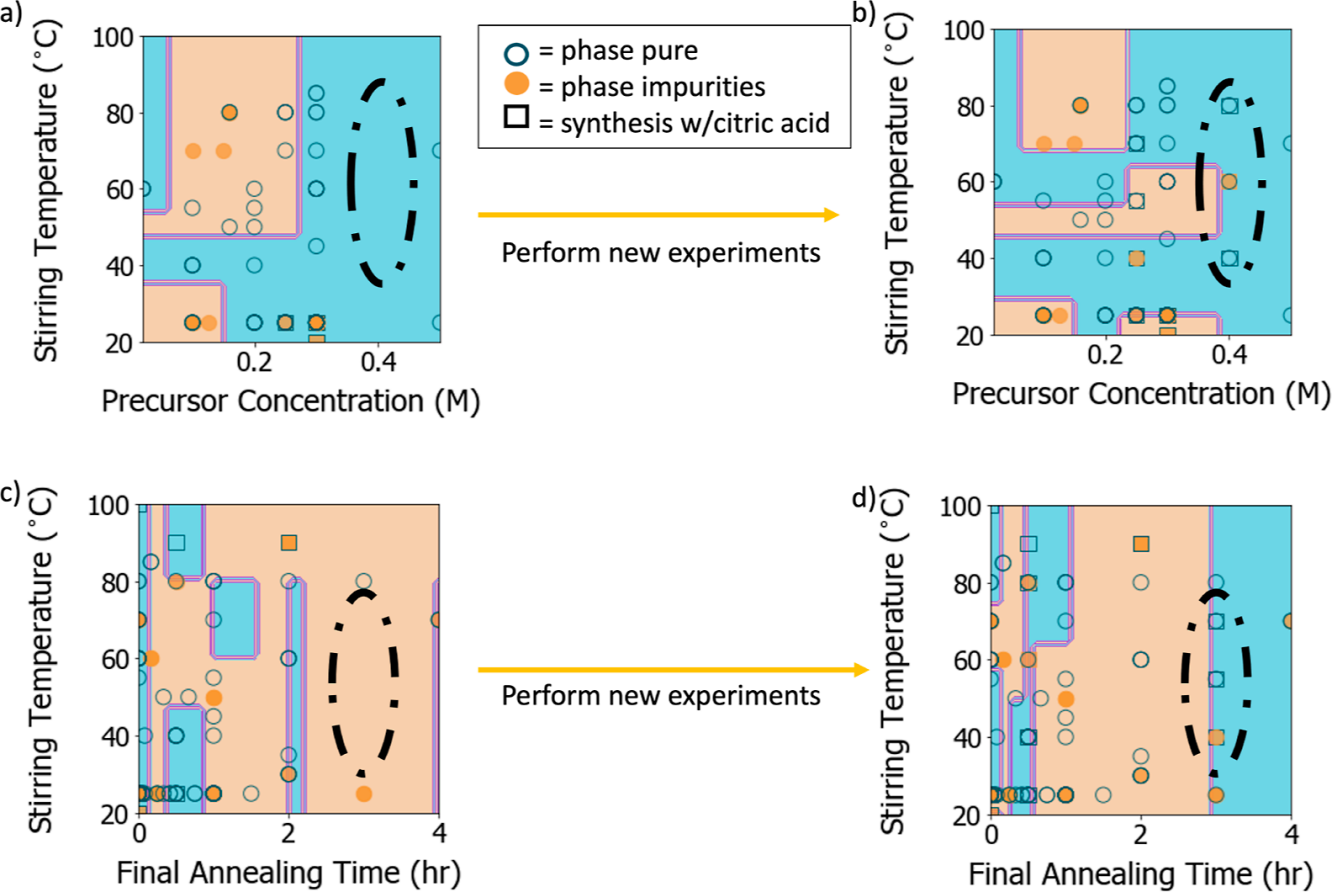
Pairwise condition distributions with targeted regions of interest (ROIs) and resulting syntheses. (a,c) Pairwise condition distributions for various synthesis conditions (stirring temperature, precursor concentration, and final annealing time) taken from the literature with ROIs for additional experiments indicated by dashed black ellipses. (b,d) Results from the suggested syntheses are then incorporated into the dataset, and the condition distributions are revisualized (b,d). Orange points and regions represent syntheses resulting in phase impurities, and blue points and regions represent phase-pure syntheses. Square points represent the use of citric acid as a chelating agent, and circles represent syntheses without citric acid.

We identified areas in the synthesis condition space that lacked data and proposed experiments to fill those gaps. With the results of these experiments, our decision tree model was then retrained to update the decision boundaries. This exploration and retraining is depicted in Figure 5. Following Figure 3b, we focused our attention on regions of synthesis conditions that are under-reported. Figure 5a,c depicts the visualizations of solution stirring temperatures with (a) solution precursor concentrations and (c) final sample annealing times reported in the literature, respectively. Regions of interest that appear unexplored are identified by dashed black ovals. For (a), we see that the use of a precursor concentration higher than the median (0.25 M) appears to improve the final phase purity of the sample, so we tested this by extrapolating to higher precursor concentrations (specifically 0.4 M, which had no data from the literature in combination with this high of a stirring temperature). For (c), we wished to fill in a gap from the literature for a relatively frequently reported condition, in this case the annealing time. We therefore suggested a set of 12 experiments that explore these two data-poor regions, while filling in the remaining conditions with median values from the rest of the dataset or values that would further interrogate the decisions made by our trained model, such as the propensity to predict phase-pure synthesis when using only 2-methoxyethanol and citric acid as the solvent and chelating agent, respectively (see Section 4.2). These suggested experiments are shown in Table 2, along with the resulting phase purity and any specific impurity phases that formed. These results were then incorporated back into the synthesis dataset, with those results highlighted in Figures 5b,d.

**Table 2:** Suggested Experimental Conditions for Exploratory Synthesis of Sol–Gel-Derived BiFeO_3_ Thin Films Based on ROIs Identified in Figure 5

exp. ID	Bi/Fe	solvent	chelating agent	conc. (M)	stir temp.	anneal temp. (°C)	anneal time (h)	impurity phase(s)
B1	1.05	2-ME		0.4	80	550	0.5	
B2					60			
B3					40			
B4	1.05	2-ME	citric acid		80	550	0.5	
B5				0.4	60			M-Bi_2_O_3_, Bi_2_Fe_4_O_9_
B6					40			
B7	1.05	2-ME		0.25	70	550	3	
B8					55			
B9					40			T-Bi_2_O_3_, Bi_2_Fe_4_O_9_, Bi_25_FeO_39_
B10	1.05	2-ME	citric acid	0.25	70	550	3	
B11					55			
B12					40			

Overall, the development of impurity phases in these experiments appears to be somewhat random. Still, we can make some observations that indicate the importance of these under-reported conditions in determining final phase purity and that highlight ambiguous effects of the inclusion of citric acid in the precursor solution. From our experiments exploring higher precursor concentration across a range of stirring temperatures, represented by Figure 5b and B1–B6 of Table 2, the only sample to form impurity phases corresponded to the intermediate stirring temperature (notably with citric acid included). This could indicate that other synthesis conditions that were not recorded (or are not typically reported) lead to both Bi-rich and Fe-rich impurity phases. We can, however, begin to see the formation of a positive correlation between precursor concentration and mixing temperature in achieving a phase-pure outcome, as seen in Figure 5b, particularly at intermediate to high stirring temperatures. Our experiments that explored longer annealing times across a range of stirring temperatures, represented by Figure 5d and B7–B12 of Table 2, show that impurity phases only form when the solution is mixed at a lower temperature. Additionally, this mix of impurity phases forms when citric acid is not incorporated into the solution. From this, we may reinforce the need to include some chelating agent in order to achieve a suitable gel prior to deposition.^40^ However, the fact that the inclusion of citric acid in B5 seemed to be the deciding factor leading to an impurity phase (compared to the phase-pure synthesis from B2 without citric acid) indicates that there remains something to be learned about the effects of citric acid on the synthesis pathway in this synthesis space. We recently investigated this effect of citric acid through in situ XRD experiments inspired by our text-mined dataset, as well as through first-principles modeling, leading to a conclusion that the use of citric acid helps mitigate the formation of the Bi_2_Fe_4_O_9_ impurity phase through the development of an intermediate bismuth subcarbonate phase.^73^

## 5 Discussion

### 5.1 Utility of Manually Text-Mined Dataset in Outcome Prediction and Variable Completion

Curating a dataset relating synthesis procedures to phase purity outcomes allowed us to train a decision tree model capable of recovering some well-known heuristics and highlighting several features that are considered important predictors for obtaining phase-pure BiFeO_3_. However, several of these important predictors (such as the Bi/Fe ratio and other precursor solution conditions) are often not reported in published synthesis descriptions, as seen from our summary of the completeness of our dataset in Section 4.1. Replication of published results is an important part of the research process and is hampered when important parameters are omitted from the reported procedure. It can be possible to impute these values through a review of other similar published procedures that include more complete descriptions. As shown by our experiments in Section 4.3, reproducing published procedures that do omit synthesis information often leads to ambiguous outcomes. Still, our reproducibility results indicate that substituting median or typical values from the literature for missing synthesis conditions can help produce results consistent with those reported, highlighting the utility of large-scale text mining for rational data gap imputation. These findings are only based on the reproduction of four syntheses reported in the literature, and a more thorough study should be conducted to make an assessment of the reproducibility of sol–gel thin film syntheses in general.

To meet our modeling goals, we curated our dataset manually. However, automated text extraction using state-of-the-art NLP tools is gaining popularity in many fields, including materials science. Despite the convenience of these methods, they are not perfect and tend to struggle with complex synthesis extraction and procedure-outcome linking. Addressing these problems is becoming more approachable with the proliferation of new large language models like GPT-3, which has been proven useful in creating materials science chatbot assistants,^32^ the extraction of complex synthesis procedures for gold nanorods,^74^ and structured information extraction of materials properties, structure, and application.^75,76^ Nonetheless, analyses performed on our manually curated dataset represent an upper limit of what can be learned through literature mining alone for the end-to-end synthesis pathways of a particular material.

### 5.2 Future Work in Experiments Inspired by Text Mining

To the best of our knowledge, there have been no published studies that perform direct experiments based on modeling and imputation from a text-mined dataset to study the impact of synthesis conditions on phase purity. Our set of suggested experiments in Table 2 is an example of how text mining the synthesis literature can be used to inform syntheses that evaluate previously under-reported conditions and their effects on phase purity. In order to increase throughput for such exploratory experiments, a combination of automatic identification of regions of interest to explore in the synthesis condition space and execution of high-throughput experiments through robotic synthesis laboratories could be implemented in the future. The results from these and additional informed experiments can also aid in constructing hypotheses regarding the effect of synthesis conditions and choices on reaction mechanisms, which can be further interrogated through first-principles models^77^ or directly investigated through in situ phase characterization.^78^

## 6 Conclusions

In this work, we constructed a text-mined dataset of sol–gel synthesis procedures and phase purity outcomes for BiFeO_3_ thin films with the goal of developing a machine learning model that predicts the presence of impurity phases as a function of synthesis conditions. The decision tree models we trained for this task achieved limited performance, with F1 scores between 0.47 and 0.52, though they confirmed a number of known heuristics for the avoidance of impurity phases, namely employing (1) an annealing temperature below ∼650 °C and (2) bismuth excess of ∼5%. Statistical analysis of the dataset revealed that many conditions are often missing from synthesis descriptions in the literature, and our modeling showed that several of these features (particularly the Bi/Fe ratio and mixing conditions) are important predictors of phase purity. Experimental attempts to replicate published syntheses while substituting typical values from the literature for these missing conditions can aid in successfully reproducing published syntheses. These values are not always obvious (such as the Bi/Fe ratio), and so these successes reflect the utility of data-driven experimental design. Some syntheses were still irreproducible after substituting missing values, highlighting the importance of reporting these vital conditions for replication, namely the concentration of the precursor solution and the Bi/Fe ratio. Finally, new exploratory experiments were inspired by visible gaps in the synthesis condition coverage of the dataset. The results of these experiments highlight several of the synthesis conditions in precursor solution preparation that affect final phase purity, namely, the precursor concentration, mixing conditions, as well as the inclusion of citric acid as a chelating agent. These experiments represent an example of how a text-mined dataset of synthesis conditions and outcomes can be used to inspire new syntheses. Because it was manually extracted and validated, our dataset can be considered as a gold standard for future ML-based synthesis learning tasks in this synthesis space or for automated text extraction models.

## Data Availability

Dataset and notebooks for data processing, modeling, and visualization are provided at https://github.com/kevcruse96/bfo-impurityphase-analysis.

## Supporting Information Available

Details on dataset structure, details on Mol2Vec implementation, frequency of substrates used, details on missing values and *k*-nearest neighbors imputation, comparison of classifier model performance, and visualization of condition distributions and phase impurity prevalence for larger subset of dataset features (PDF)
