# Supplementary material for: Text Mining the Literature to Inform Experiments and Rationalize Impurity Phase Formation for BiFeO3
Source: Chem Mater. 2023 Dec 29;36(2):772–85. doi: 10.1021/acs.chemmater.3c02203 (PMC10809418; doi:10.1021/acs.chemmater.3c02203)
Supplement: Supplementary file 1 — cm3c02203_si_001.pdf [file cm3c02203_si_001.pdf]

Text-mining the literature to inform experiments and rationalize impurity phase formation for $BiFeO_3$

Kevin Cruse[1, 2], Viktoriia Baibakova[3], Maged Abdelsamie[4,5], Kootak Hong[6,7], Christopher J. Bartel[1,2,8], Amalie Trewartha[1,2,9], Anubhav Jain[3], Carolin M. Sutter-Fella[10], and Gerbrand Ceder[1,2,*]

[1]Department of Materials Science & Engineering, University of California, Berkeley, CA 94720, USA

[2]Materials Sciences Division, Lawrence Berkeley National Laboratory, Berkeley, CA 94720, USA

[3]Energy Technologies Area, Lawrence Berkeley National Laboratory, Berkeley, CA 94720, USA

[4]Material Science and Engineering Department, King Fahd University of Petroleum and Minerals (KFUPM), Dhahran 31261, Saudi Arabia

[5]Interdisciplinary Research Center for Intelligent Manufacturing and Robotics, KFUPM, Dhahran 31261, Saudi Arabia

[6]Chemical Sciences Division, Lawrence Berkeley National Laboratory, Berkeley, CA 94720, USA

[7]Department of Materials Science and Engineering, Chonnam National University, Gwangju 61186, Republic of Korea

[8]Department of Chemical Engineering and Materials Science, University of Minnesota, Minneapolis, MN 55455, USA

[9]Energy and Materials, Toyota Research Institute, Los Altos, CA 94022 USA

[10]Molecular Foundry Division, Lawrence Berkeley National Laboratory, Berkeley, CA 94720, USA

[*]Corresponding author: Gerbrand Ceder (gceder@berkeley.edu)

Supplementary Information

S1 Extraction Details

Finer details on all extracted entities for the text-mined sol-gel-derived $BiFeO_3$ thin-film synthesis dataset are given in Table S1. Each entity was extracted manually and numeric quantities were converted to the appropriate unit if necessary. Entities were left blank for information that was not provided for a given synthesis.

Feature	Type	Description
recipe_id	`int`	ID for individual synthesis procedure
url	`string`	URL to article
doi	`string`	DOI for article
source_Bi	`string`	Bismuth source
source_Bi_amount_g	`float`	Amount of bismuth source in grams
source_Fe	`string`	Iron source
source_Fe_amount_g	`float`	Amount of iron source in grams
separate_hydrolysis	`boolean`	Indicator for whether Bi and Fe sources were dissolved separately
solvent1	`string`	Name of first type of solvent
solvent1_amount_ml	`float`	Amount of first type of solvent in mL
solvent2	`string`	Name of second type of solvent, if present
solvent2_amount_ml	`float`	Amount of second type of solvent in mL
solv1_solv2_ratio	`float`	Ratio of 2 solvents if given
chelating_agent1	`string`	Name of first type of chelating agent
chelating_agent1_amount_ml	`float`	Amount of first type of chelating agent in mL
chelating_agent2	`string`	Name of second type of chelating agent
chelating_agent2_amount_ml	`float`	Amount of second type of chelating agent in mL
chelating_agent3	`string`	Name of third type of chelating agent
chelating_agent3_amount_ml	`float`	Amount of third type of chelating agent in mL
solv_chel_ratio	`float`	Ratio between solvent and chelating agent if any are given
dehydrating_agent1	`string`	Name of first type of dehydrating agent
dehydrating_agent1_amount_ml	`float`	Amount of first type of dehydrating agent in mL
dehydrating_agent2	`string`	Name of second type of dehydrating agent
dehydrating_agent2_amount_ml	`float`	Amount of second type of dehydrating agent in mL
solv_dehydr_ratio	`float`	Ratio between solvent and dehydrating agent if any are given
surfactant	`string`	Name of surfactant
surfactant_amount	`float`	Amount of surfactant in mL
solv_surfactant_ratio	`float`	Ratio between solvent and surfactant if given
precursor_concentration	`float`	Concentration of metal nitrates in precursor solution
pH	`float`	pH of precursor solution

substrate	`string`	Type of substrate for spin-coating
substrate_orientation	`string`	Orientation of top layer of substrate
stirring_time_hr	`float`	Precursor solution mixing time in hours
stirring_temp_degC	`float`	Precursor solution mixing temperature in °C
age_days	`float`	Number of days precursor solution is aged
age_temp_degC	`float`	Temperature at which precursor solution is aged
low_coating_time_sec	`float`	Slower spin-coating time in seconds if two-step
low_coating_rpm	`float`	Slower spin-coating rate in rpm if two-step
high_coating_time_sec	`float`	Faster spin-coating time in seconds
high_coating_rpm	`float`	Faster spin-coating rate in rpm
dry_time_min	`float`	Spin-coated layer drying time in minutes
dry_degC	`float`	Spin-coated layer drying temperature in °C
layer_prebake_time_min	`float`	Spin-coated layer pyrolysis time in minutes
layer_prebake_temp_degC	`float`	Spin-coated layer pyrolysis temperature in °C
layer_annealing_time_min	`float`	Spin-coated layer annealing time in minutes
layer_annealing_degC	`float`	Spin-coated layer annaling temperature in °C
layers	`int`	Number of layers spin-coated for thin film
final_prebake_time_min	`float`	Prebake time after all layers spin-coated in minutes
final_prebake_degC	`float`	Prebake temperature after all layers spin-coated in °C
final_annealing_time_hr	`float`	Final annealing time after all layers spin-coated in hours
final_annealing_degC	`float`	Final annealing temperature after all layers spin-coated in °C
atmosphere	`string`	Type of environment used for annealing (often air, O_2, or N_2)
reported_impurities	`list of strings`	List of any reported secondary phases

Table S1: Fine details on extracted synthesis features

S2 Mol2Vec Embeddings

S2.1 Flowchart to Acquire Mol2Vec Embeddings

Figure S1 depicts a flowchart for acquiring chemical embeddings from the Mol2Vec model. First, chemicals are identified by their SMILES strings which are used to collect the list of Morgan fingerprint identifiers for chemical substructures. Each substructure has been given a 300-dimensional embedding from a pre-trained model trained on amino acid structures. The dimension of this collection of substructure embeddings is then reduced using principal component analysis. The resulting coordinates can then be used to create vector summations of each substructure to form a molecule, similar to the vector summation construction of words into phrases from the Word2Vec model.

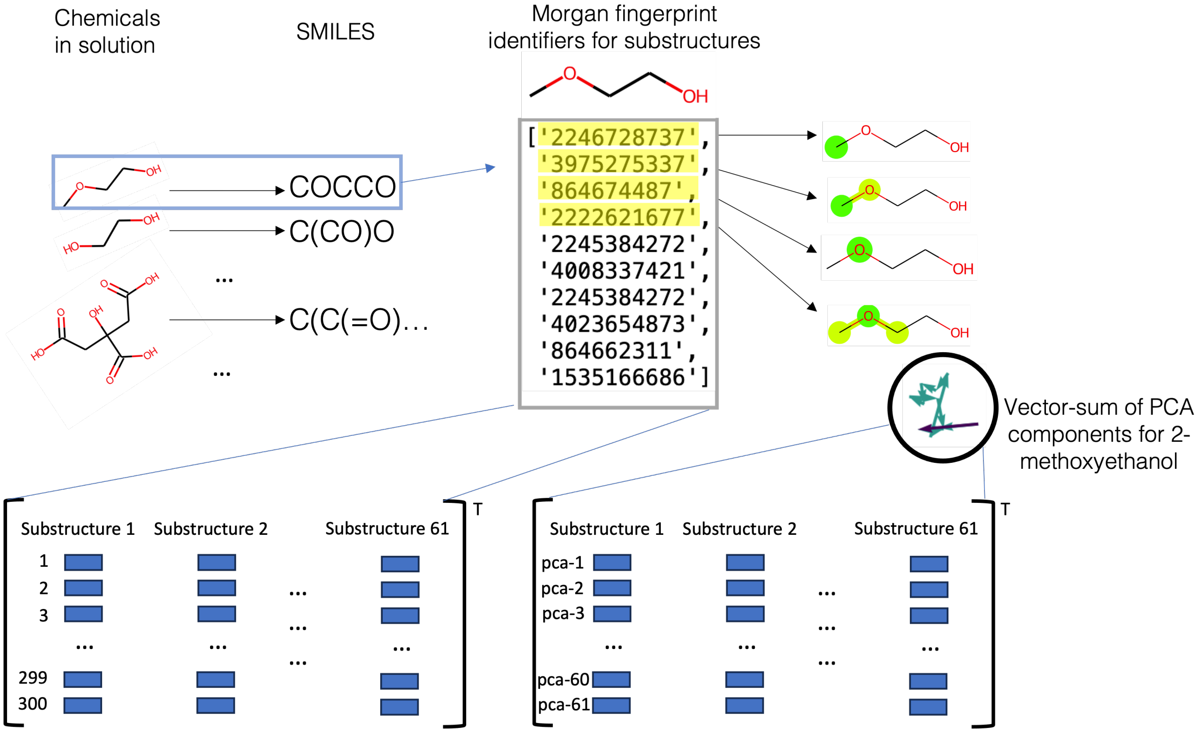

Figure S1: **Flowchart for acquiring chemical embeddings from Mol2Vec.**

S2.2 Mol2Vec Chemical Embedding Convergence

The convergence plot for average change in cosine-similarity between principle components of chemical embeddings is shown in Figure S2. The plot shows convergence at around 20-30 principle components. The cosine similarities for all possible reagents when considering 30 principle components is given in Figure S3.

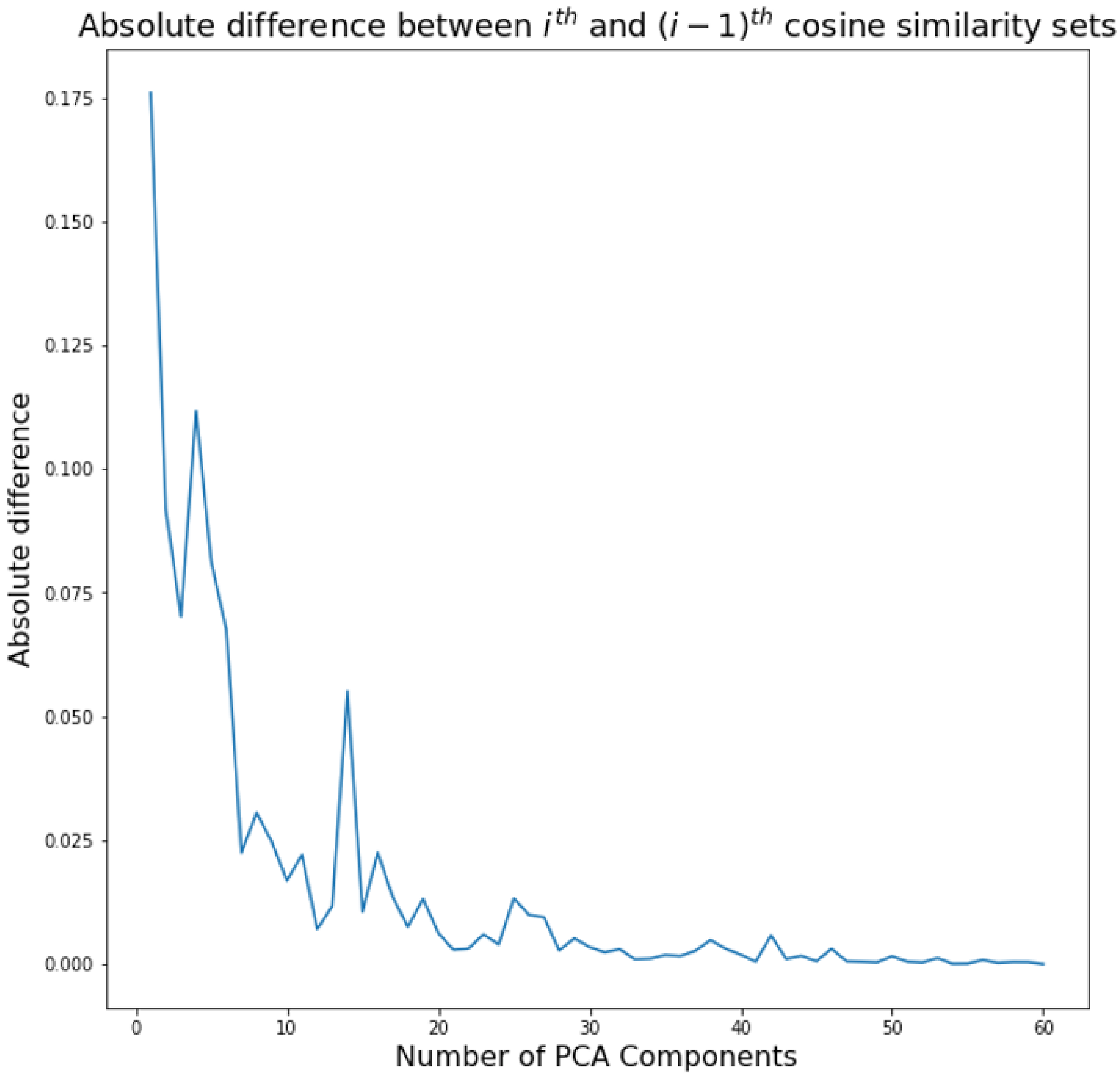

Figure S2: **Convergence plot for chemical embedding PCA cosine similarity.** Average change in cosine similarity between principle components of chemical embeddings for all chemical species across possible PCA vector space.

	2-methoxyethanol	ethylene glycol	trimethylene glycol	ethanol	acetic acid	citric acid	acetic anhydride	ethanolamine	diethanolamine	nitric acid	ethylenediamine	ammonium hydroxide	acetone	acetylacetone	polyvinylpyrrolidone	nn_dimethylformamide
2-methoxyethanol	1.000000	0.688559	0.741374	0.664697	0.151051	0.055816	0.252859	0.519350	0.726575	-0.334327	0.239881	-0.112377	0.292642	0.188050	-0.118413	0.022088
ethylene glycol	0.688559	1.000000	0.967699	0.636062	0.142188	0.223411	-0.066650	0.726870	0.852182	-0.168245	0.310476	-0.105997	0.017447	0.011576	-0.146682	-0.144741
trimethylene glycol	0.741374	0.967699	1.000000	0.669532	0.156520	0.230341	-0.021770	0.770629	0.931928	-0.225310	0.393512	-0.116509	0.065838	0.083750	-0.161581	-0.151938
ethanol	0.664697	0.636062	0.669532	1.000000	0.430622	0.288150	0.207577	0.491515	0.619447	-0.269972	0.237871	-0.065279	0.330417	0.212536	-0.139094	0.016497
acetic acid	0.151051	0.142188	0.156520	0.430622	1.000000	0.680981	0.645877	0.164219	0.224850	-0.258543	0.128392	-0.132584	0.786967	0.669741	-0.102666	0.077752
citric acid	0.055816	0.223411	0.230341	0.288150	0.680981	1.000000	0.319697	0.245948	0.275628	-0.206768	0.185017	-0.169966	0.417054	0.469078	-0.290608	-0.191085
acetic anhydride	0.252859	-0.066650	-0.021770	0.207577	0.645877	0.319697	1.000000	-0.072100	0.030384	-0.260641	-0.053433	-0.207159	0.869783	0.755194	0.007508	0.162707
ethanolamine	0.519350	0.726870	0.770629	0.491515	0.164219	0.245948	-0.072100	1.000000	0.730650	-0.273100	0.878511	-0.105985	-0.028195	0.067879	-0.260654	-0.155054
diethanolamine	0.726575	0.852182	0.931928	0.619447	0.224850	0.275628	0.030384	0.730650	1.000000	-0.246066	0.418530	-0.119331	0.151870	0.176730	-0.182249	-0.154806
nitric acid	-0.334327	-0.168245	-0.225310	-0.269972	-0.258543	-0.206768	-0.260641	-0.273100	-0.246066	1.000000	-0.260972	0.078828	-0.248229	-0.262659	-0.154018	-0.229814
ethylenediamine	0.239881	0.310476	0.393512	0.237871	0.128392	0.185017	-0.053433	0.878511	0.418530	-0.260972	1.000000	-0.072965	-0.051161	0.085900	-0.258745	-0.113932
ammonium hydroxide	-0.112377	-0.105997	-0.116509	-0.065279	-0.132584	-0.169966	-0.207159	-0.105985	-0.119331	0.078828	-0.072965	1.000000	-0.182798	-0.264654	-0.228678	-0.167701
acetone	0.292642	0.017447	0.065838	0.330417	0.786967	0.417054	0.869783	-0.028195	0.151870	-0.248229	-0.051161	-0.182798	1.000000	0.801219	0.080010	0.214019
acetylacetone	0.188050	0.011576	0.083750	0.212536	0.669741	0.469078	0.755194	0.067879	0.176730	-0.262659	0.085900	-0.264654	0.801219	1.000000	-0.091285	0.061581
polyvinylpyrrolidone	-0.118413	-0.146682	-0.161581	-0.139094	-0.102666	-0.290608	0.007508	-0.260654	-0.182249	-0.154018	-0.258745	-0.228678	0.080010	-0.091285	1.000000	-0.006427
nn_dimethylformamide	0.022088	-0.144741	-0.151938	0.016497	0.077752	-0.191085	0.162707	-0.155054	-0.154806	-0.229814	-0.113932	-0.167701	0.214019	0.061581	-0.006427	1.000000

Figure S3: Table showing pairwise cosine difference values between vector representations of each chemical using mol2vec algorithm.

S3 Frequency of Substrates

Figure S4 depicts the frequency of specific substrates used in the text-mined dataset.

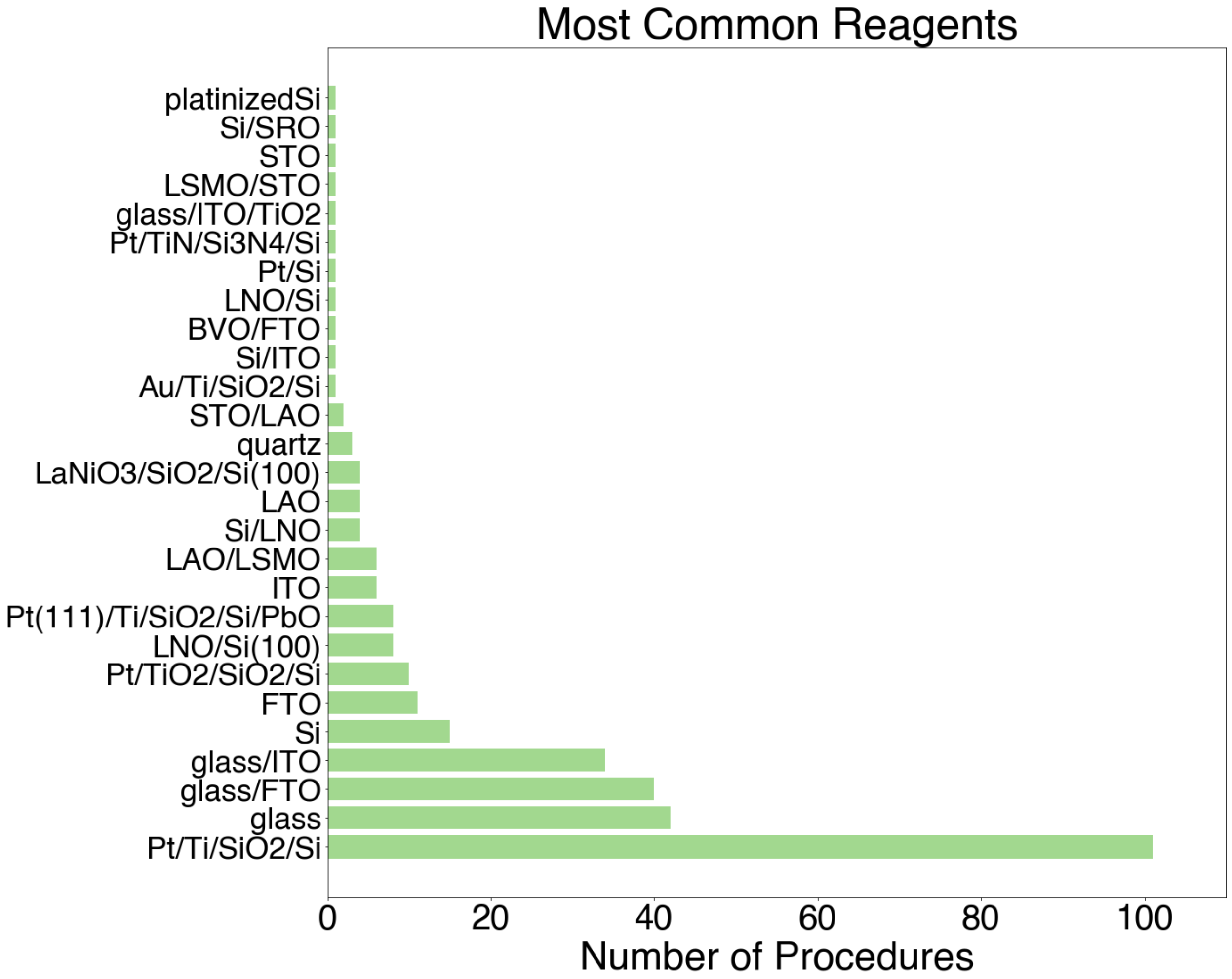

Figure S4: **Frequency of substrates**: Frequency of specific substrates used in text-mined dataset.

S4 Missing Value Analysis and Imputation

S4.1 Condition Value Heatmap

Figure S5 depicts the range of values employed for all condition in the synthesis dataset, as well as the frequency of omitted values for each condition.

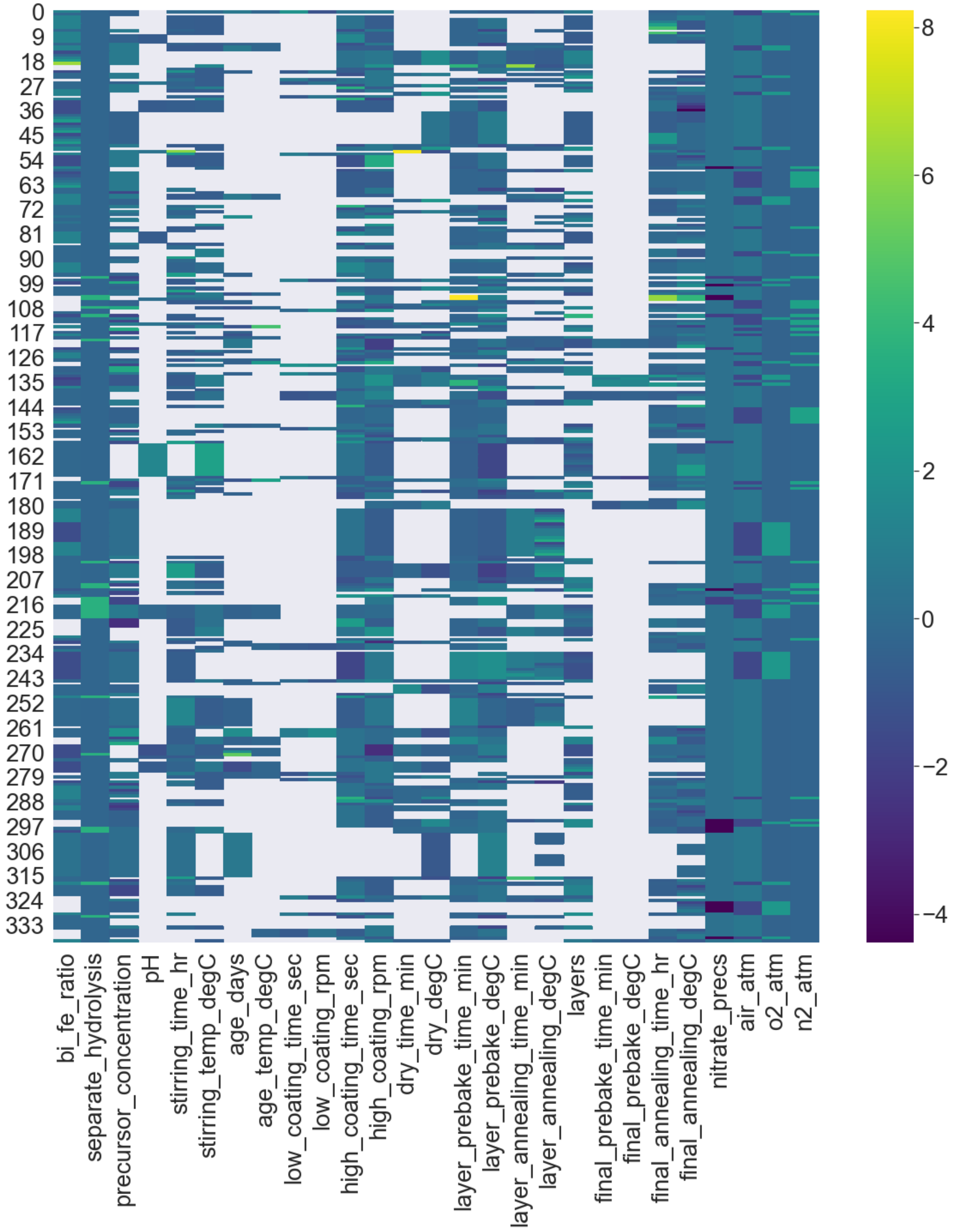

Figure S5: **Synthesis condition heatmap**: Individual syntheses from the text-mined dataset (rows) encoded by their conditions (columns). The heat in each cell represents the deviation from the mean value for that condition in that particular procedure. Grey values represent missing values.

S4.2 k-Nearest Neighbors Imputation

We tested the ability for k-nearest neighbors missing value imputation to correctly impute values by randomly masking values for features of interest (Bi:Fe ratio, precursor, concentration, and mixing time / temperature, in alignment with the missing values analysis from Figure 3) and comparing the imputed value with the true value. We first acquired a subset of 110 rows from our dataset for which none of the values of the aforementioned features were missing. Then we randomly masked 20% of each of those feature values so that they would be labeled as "missing". We then performed kNN imputation on a scaled version of the dataset (since nearest neighboring data points are identified using Euclidean distance) and compared with the dataset of true values to find the frequency of exact matches. We found that with 95 total values masked and imputed we achieved perfect imputation for 35 of the values when k=5 nearest neighbors, 51 values when k=3, and 72 values when k=1. There is a risk of overfitting when using small values for k; thus, we moved forward with $k = 5$ so that the imputer is not resigned to the local structure of the data point.

S5 Classifier Model Comparison

We provide details on a comparison in performance evaluation as well as details on hyperparameter-tuning for four different classifier algorithms (decision trees, random forest, extra trees, and XGBoost).

S5.1 Hyperparameter Grids

The following lists the specific hyperparameters and values considered in cross-validation for each classifier algorithm. For the decision tree classifier we used sklearn's GridSearchCV module and for the random forest, extra trees, and XGBoost classifiers we used sklearn's RandomizedSearchCV module. Cross-validation was stratified, by 5 folds, 10 repeats, and the same random state set for each training. The F1 score (with phase impurity formation being the positive class) was used to determine the best estimator from cross-validation. For randomized search cross-validation, 50 iterations were used for random forest and extra trees and 500 iterations were used for XGBoost.

Decision Tree

- `criterion: ["gini", "entropy"]`
- `splitter: ["best", "random"]`
- `max_depth: range(10,21)`
- `min_samples_split: range(2, 4)`
- `min_samples_leaf: range(1, 3)`
- `random_state: [2**r for r in range(7, 17)]`

- class_weight: ['balanced']

Random Forest

- criterion: ["gini"]
- n_estimators: [100, 200, 300, 400, 500, 1000]
- max_depth: range(10, 50)
- max_features: ['auto', 'sqrt', None]
- min_samples_split: [2, 5, 10]
- min_samples_leaf: [1, 2, 4]
- bootstrap: [True, False]
- class_weight: ["balanced"]

Extra Trees

- criterion: ["gini"]
- n_estimators: [100, 200, 300, 400, 500, 1000]
- max_depth: range(10, 50)
- max_features: ['auto', 'sqrt', None]
- min_samples_split: [2]
- min_samples_leaf: [1]
- bootstrap: [True, False]
- class_weight: ["balanced"]

XGBoost

- n_estimators: [50, 60, 75, 90, 100, 125, 150, 200, 300]
- gamma: [0.5, 1, 1.5, 2, 5, 10]
- learning_rate: [0.01, 0.02, 0.05, 0.07, 0.1, 0.2]
- subsample: [0.5, 0.6, 0.7, 0.8]
- colsample_bytree: [0.5, 0.6, 0.7, 0.8, 1.0]
- max_depth: range(10,50)
- reg_alpha: [0, 0.1, 0.2, 0.5, 0.7, 1.0, 1.5, 2, 2.5, 3, 5, 7, 10, 100]
- reg_lambda: [0, 0.1, 0.2, 0.5, 0.7, 1.0, 1.5, 2, 2.5, 3, 5, 7, 10, 100]

S5.2 Performance Comparison Between Algorithms

Tables S2-S5 show the averages and standard deviations for several evaluation metrics for sets of 10 trained models within each algorithm type, separated by the different imputation and classification task frameworks. Evaluation metrics include the F1 score (where the positive class corresponds to forming phase impurities), precision, recall, Mathew's correlation coefficient (MCC), normalized false positives (calculated as the number of false positives divided by the total number of positive cases, similar to Type I Error), normalized false negatives (calculated as the number of false negatives divided by the total number of negative cases, similar to Type II Error), and the macro-averaged F1 score (calculated as the average of the class-wise F1 scores).

**MMVI Binary**	Decision Tree	Random Forest	Extra Trees	XGBoost
F1	0.51 ± 0.08	0.50 ± 0.06	0.50 ± 0.07	0.45 ± 0.15
Precision	0.61 ± 0.13	0.68 ± 0.16	0.74 ± 0.12	0.75 ± 0.17
Recall	0.45 ± 0.09	0.41 ± 0.07	0.38 ± 0.05	0.35 ± 0.12
MCC	0.38 ± 0.11	0.40 ± 0.10	0.43 ± 0.10	0.38 ± 0.11
Normalized FP	0.39 ± 0.13	0.32 ± 0.16	0.26 ± 0.12	0.25 ± 0.17
Normalized FN	0.18 ± 0.02	0.19 ± 0.02	0.19 ± 0.02	0.20 ± 0.03
Macro F1	0.68 ± 0.05	0.68 ± 0.04	0.69 ± 0.04	0.66 ± 0.08

Table S2: Average evaluation metrics and standard deviations for different algorithms trained on median value-imputed dataset for binary classification task.

**MMVI Multilabel**	Decision Tree	Random Forest	Extra Trees	XGBoost
F1	0.49 ± 0.06	0.52 ± 0.11	0.48 ± 0.12	0.45 ± 0.08
Precision	0.62 ± 0.16	0.62 ± 0.16	0.81 ± 0.13	0.85 ± 0.10
Recall	0.42 ± 0.08	0.46 ± 0.10	0.34 ± 0.10	0.31 ± 0.07
MCC	0.37 ± 0.09	0.39 ± 0.15	0.44 ± 0.13	0.43 ± 0.08
Normalized FP	0.38 ± 0.16	0.38 ± 0.16	0.19 ± 0.13	0.15 ± 0.10
Normalized FN	0.18 ± 0.02	0.18 ± 0.03	0.20 ± 0.03	0.20 ± 0.02
Macro F1	0.67 ± 0.04	0.68 ± 0.07	0.68 ± 0.07	0.67 ± 0.04

Table S3: Average evaluation metrics and standard deviations for different algorithms trained on median value-imputed dataset for multilabel classification task.

***k*NNMVI Binary**	Decision Tree	Random Forest	Extra Trees	XGBoost
F1	0.50 ± 0.06	0.52 ± 0.06	0.51 ± 0.06	0.42 ± 0.12
Precision	0.56 ± 0.06	0.72 ± 0.12	0.74 ± 0.10	0.72 ± 0.16
Recall	0.46 ± 0.09	0.41 ± 0.04	0.39 ± 0.05	0.32 ± 0.12
MCC	0.35 ± 0.14	0.43 ± 0.09	0.43 ± 0.07	0.35 ± 0.12
Normalized FP	0.44 ± 0.13	0.28 ± 0.12	0.26 ± 0.10	0.28 ± 0.16
Normalized FN	0.18 ± 0.03	0.19 ± 0.02	0.19 ± 0.01	0.21 ± 0.03
Macro F1	0.67 ± 0.07	0.69 ± 0.04	0.69 ± 0.04	0.64 ± 0.07

Table S4: Average evaluation metrics and standard deviations for different algorithms trained on k-nearest neighbors-imputed dataset for binary classification task.

***k*NNMVI Multilabel**	Decision Tree	Random Forest	Extra Trees	XGBoost
F1	0.47 ± 0.05	0.51 ± 0.05	0.45 ± 0.08	0.50 ± 0.07
Precision	0.62 ± 0.12	0.77 ± 0.17	0.77 ± 0.17	0.90 ± 0.09
Recall	0.39 ± 0.06	0.40 ± 0.06	0.33 ± 0.08	0.35 ± 0.07
MCC	0.36 ± 0.07	0.44 ± 0.09	0.40 ± 0.11	0.49 ± 0.07
Normalized FP	0.38 ± 0.12	0.23 ± 0.17	0.23 ± 0.17	0.10 ± 0.09
Normalized FN	0.18 ± 0.01	0.18 ± 0.01	0.20 ± 0.02	0.19 ± 0.02
Macro F1	0.66 ± 0.03	0.69 ± 0.03	0.66 ± 0.05	0.70 ± 0.04

Table S5: Average evaluation metrics and standard deviations for different algorithms trained on k-nearest neighbors-imputed dataset for multilabel classification task.

S6 Pairwise Synthesis Condition Visualization

Full factorial visualization of selected condition pairs for sol-gel-derived $BiFeO_3$ thin-film synthesis are shown in Figure S6.

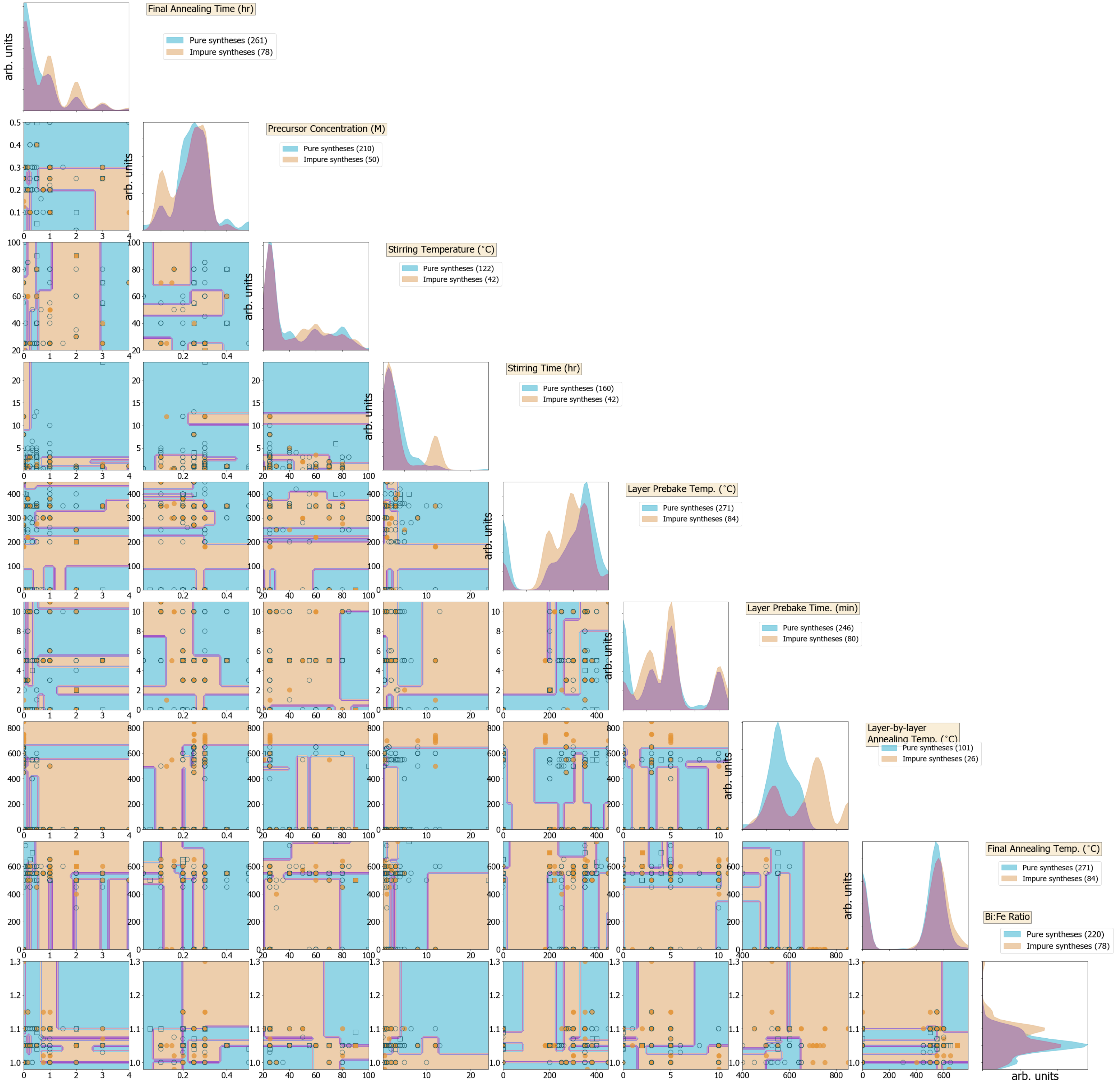

Figure S6: Full factorial visualization of pairs of synthesis conditions
